# Hik28-dependent and Hik28-independent ABC transporters were revealed by proteome-wide analysis of ΔHik28 under combined stress

**DOI:** 10.1186/s12860-022-00421-w

**Published:** 2022-07-06

**Authors:** Pavinee Kurdrid, Rayakorn Yutthanasirikul, Sirilak Saree, Jittisak Senachak, Monpaveekorn Saelee, Apiradee Hongsthong

**Affiliations:** 1 Biosciences and System Biology Team, National Center for Genetic Engineering and Biotechnology, National Science and Technology Development Agency at King Mongkut’s University of Technology Thonburi, Bangkok, 10150 Thailand; 2 Pilot Plant Development and Training Institute, King Mongkut’s University of Technology Thonburi, Bangkok, 10150 Thailand

**Keywords:** Metabolic pathway, Stress response, Response mechanism, Deletion mutant, Genome and Cyanobacteria

## Abstract

*Synechocystis* histidine kinase, Sll0474: Hik28, a signal protein in a two-component signal transduction system, plays a critical role in responding to a decrease in growth temperature and is also involved in nitrogen metabolism. In the present study, under combined stress from non-optimal growth temperature and nitrogen depletion, a comparative proteomic analysis of the wild type (WT) and a deletion mutant (MT) of *Synechocystis* histidine kinase, Sll0474: Hik28, in a two-component signal transduction system identified the specific groups of ABC transporters that were Hik28-dependent, e.g., the iron transporter, and Hik28-independent, e.g., the phosphate transporter. The iron transporter, AfuA, was found to be upregulated only in the WT strain grown under the combined stress of high temperature and nitrogen depletion. Whereas, the expression level of the phosphate transporter, PstS, was increased in both the WT and MT strains. Moreover, the location in the genome of the genes encoding Hik28 and ABC transporters in *Synechocystis sp.* PCC6803 were analyzed in parallel with the comparative proteomic data. The results suggested the regulation of the ABC transporters by the gene in a two-component system located in an adjacent location in the genome.

**Supplementary Information:**

The online version contains supplementary material available at 10.1186/s12860-022-00421-w.

## Introduction

In *Spirulina*, the changes in the protein profile at a low temperature (22 °C) were examined at the subcellular level, and it was reported that the proteins involved in the two-component response system, DNA repair, chaperones and nitrogen uptake play an important role in the response of *Spirulina* to low-temperature stress [1, 2]. Moreover, a proteomic analysis of the cyanobacterium *Synechocystis* sp. PCC 6803 was performed in the optimal range of growth temperatures, namely, 32–35 °C, and higher in the thermal tolerance range (42 °C). Sixty-five proteins in the categories of heat shock proteins, protein biosynthetic machinery, amino acid biosynthetic enzymes, components of the light and dark photosynthetic apparatus, and energy metabolism were differentially expressed within 1 h after heat shock [3, 4].

Two-component systems (TCSs), consisting of a sensor histidine kinase and a response regulator, play a crucial role in the stress response mechanism. These regulatory systems mediate acclimatization to various environmental changes by linking environmental signals to gene expression. One of the sensor histidine kinase proteins found in cyanobacteria is Hik2, which is a homolog of the chloroplast sensor kinase (CSK) [5]. The protein is involved in redox regulation of chloroplast gene expression in plants and algae during changes in light quality. Moreover, Hik2 shows redundancy with Hik33, which is responsible for sensing osmotic and low-temperature stress [6]. It was proposed that Hik2 and Hik33 are involved in the resistance of PS II to environmental stresses. Furthermore, Hik33 was reported to regulate the expression of cold-inducible genes for membrane lipid biosynthesis in *Synechocystis*, whereas *Synechocystis* Hik34 was found to be an essential component for long-term high-temperature adaptation [7, 8]. The *Synechocystis* wild-type strain was able to recover after 24 h of cultivation at 44 °C, while the ΔHik34 mutant strain was resistant to heat stress only within the first hour, and the mutant could not recover after 24 h of exposure to high-temperature treatment [9].

In *Arthrospira platensis* strain C1, the combined stress of nitrogen depletion and high temperature was studied, and it was found that photosynthetic activity was reduced by more than half under these conditions compared to stress-free conditions. Moreover, reductions in biomass and total protein were reported under combined stress. The accumulation of linoleic acid (C18:2) and a decrease in γ-linolenic acid within 24 h of stress exposure were observed, together with an increasing level of carbohydrate content [10].

In our previous study, two proteins of *Arthrospira platensis* C1, namely, the multisensor histidine kinase SPLC1_S041070 (Hik28) and glutamate synthase, were found to interact in a yeast two-hybrid system. Due to the lack of a specific gene manipulation system and gene transformation in *Arthrospira*, a deletion mutant of *Synechocystis* Hik28, sll0474, which is a homolog of SPLC1_S041070, was constructed and grown under nitrogen depletion and a combination of nitrogen depletion and temperature stress, either 16 °C (low temperature) or 45 °C (high temperature). The fatty acid composition of the WT and MT strains under nitrogen depletion and combined stress was analyzed by using gas chromatography (GC). The data showed the accumulation of C16:1^Δ9^. Moreover, the chlorophyll content and O_2_ evolution rate were decreased drastically under nitrogen depletion and combined stress in both the WT and MT strains, although the rates of the MT cells were lower than those of the WT [11]. However, the analysis of the proteome-wide effect of Hik28 deletion is still required to indicate the possible role of Hik28 under combined stress. Thus, in the present study, proteomic analyses of the WT and MT (∆Hik28) strains were carried out under temperature stress and combined stress by using liquid chromatography–tandem mass spectrometry (LC–MS/MS). The response mechanism regulated by Hik28 and its subsequent effect on metabolic pathways could be elucidated by comparative analysis of the proteomic data from the WT and MT strains under both forms of stress together with in-depth analysis of the protein–protein interaction network and gene location in the genome by using available databases.

## Materials and methods

### Cell growth and conditions

The construction of the Hik28, Sll0474, deletion mutant (MTΔHik28) was described in a previous study [11], and oligonucleotide primers for the construction of the ΔHik28 mutant in *Synechocystis* sp. PCC6803 are shown in Suppl. Fig. 1 and Suppl. Table 1. Cultures of the *Synechocystis* sp. PCC 6803 wild type and Hik28-deletion mutant were grown in BG-11 medium under a light intensity of 70 μEm^-2^s^-1^ at 30 °C until the optical density at 730 nm reached 0.8-0.9 (mid-log phase) and then harvested by centrifugation at 8,000 rpm for 10 min. The WT and MT cells in the control treatment were washed in normal BG-11 medium and subsequently resuspended in normal BG-11 medium (control treatment). For the nitrogen stress condition, the WT and MT cells were washed in nitrogen-free BG-11 medium and subsequently resuspended in nitrogen-free BG-11 medium (nitrogen-free treatment). Then, the WT and MT cultures receiving the control treatment (normal BG-11) and the nitrogen-free treatment (nitrogen-free BG-11 medium) were grown under 3 temperature conditions: optimal temperature (30 °C), low temperature (16 °C) and high temperature (45 °C). The cultures grown under each experimental condition were collected at 0, 1 and 24 h for further analysis.

In the case of *Arthrospira platensis* strain C1, the cells were grown in Zarrouk’s medium at the optimal temperature of 35 °C under a light intensity of 100 μEm^−2^s^−1^. The culture was grown at 35 °C until mid-log phase (when optical density at 560 nm reached 0.4-0.6) and then harvested by centrifugation. Genomic DNA was extracted from the cells by using a Genomic DNA Purification Kit (Promega, USA).

### Construction and yeast two-hybrid assays

*Arthrospira* SPLC1_S041070 (multisensor hybrid histidine kinase) was cloned into pGBKT7, and SPLC1_S630120 (glutamine synthetase) and SPLC1_S240970 (nitrogen regulatory protein P-II) were cloned into the pGAT7 vector. *Arthrospira* genomic DNA was used as a template for PCR amplification of these genes by using oligonucleotide primers (Suppl. Table 2), and the PCR products were cloned into pGBKT7 and pGAT7 vectors. Then, the constructed vectors were transformed into *Saccharomyces cerevisiae*.

Protein–protein interactions were examined by using a yeast two-hybrid system. Bait and prey proteins were cloned into pGBKT7 and pGAT7 vectors, respectively (Suppl. Fig. 2). The positive control (pGBKT7-p53 vector), negative control (pGBKT7-Lam) and bait protein in pGBKT7 were transformed into *Saccharomyces cerevisiae* strain Y2HGold. The control vector pGADT7-T and prey protein in the pGAT7 vector were transformed into the Y187 strain (Clontech, USA). Y2HGold and Y187 cells were mated in 300 μl of 2xYPDA broth at 30 °C and shaken at 200 rpm for 16-18 h. The yeast mating cultures were spread onto SD/−Leu/−Trp/X-α-gal/AbA dropout (DDO/X/A) plates and incubated at 30 °C for 3 days. The blue colonies were selected, streaked onto SD/−Ade/−His/−Leu/−Trp/X-α-gal/AbA dropout (QDO/X/A) plates, and incubated at 30 °C for 3–5 days. Subsequently, bait and prey protein plasmids were switched into the yeast strains Y187 and Y2HGold, respectively, to confirm the specific interactions between bait and prey proteins (Suppl. Table 2).

### Growth and chlorophyll measurement

The cell growth of the *Synechocystis* sp. PCC6803 wild type and ∆Hik28 mutant was examined by OD_730_ measurement for the growth curve and OD_665_ measurement for chlorophyll content at 0, 1 and 24 h. Chlorophyll was extracted from the cells using 100% methanol. Chlorophyll a concentrations were calculated according to the following equation [12, 13].

$$\mathrm{Chlorophyll}-\mathrm{a}\left(\mu \mathrm{g}/\mathrm{ml}\right)={12.9447}^{\ast}\left(\mathrm{OD}_{665}\right)^\ast \mathrm{dilution}\ \mathrm{of}\ \mathrm{cell}$$

$$\text{Absorption coefficient of }Synechocystis=12.9447$$

### Oxygen evolution measurement

To examine oxygen evolution by using a Clark-type oxygen electrode, the cell suspension at a chlorophyll concentration of 2.5 μg/ml was measured by using a light illumination intensity of 160 μEm^-2^s^-1^ at 30 °C in BG-11 medium (+NO_3_ and -NO_3_). The O_2_ evolution rate was measured in three independent experiments.

$${\mathrm{O}}_2\ \mathrm{evolution}\left(\mu \mathrm{mol}\ {\mathrm{O}}_2{\mathrm{mg}}^{-1}\mathrm{Chl}\ {\mathrm{h}}^{-1}\right)=\left(\mathrm{slope}\times 60\ \min\ {\mathrm{h}}^{-1}\times 1000\right)/\left(2.5\ \mathrm{mg}\ \mathrm{Chl}\ {\mathrm{L}}^{-1}\right)$$

### Protein preparation

The WT and ΔHik28 cells were harvested by centrifugation at 8,000 rpm after being cultured at 30 °C and 45 °C for 0, 1 and 24 h following exposure to the designated stress conditions; the cells were then washed in 5 mM HEPES (pH 7.0). The cell pellets were dissolved in lysis buffer (20 mM ammonium bicarbonate, 6 M urea, 2 M thiourea, and one tablet of protease inhibitor). The cells were lysed by sonication on ice, and the supernatants were separated by centrifugation at 8,000 rpm at 4 °C for 30 min. Subsequently, 1 volume of the supernatant was diluted with 9 volumes of absolute ethanol and incubated at -20 °C for 16 h. Then, the protein was precipitated by centrifugation at 8,000 rpm at 4 °C for 30 min. The protein pellets were washed with absolute ethanol and then dissolved with 20 mM ammonium bicarbonate pH 8.5. The protein concentration was measured by using a 2D-Quant kit (GE Healthcare Life Sciences USA).

### Protein digestion

Protein treatment was done with reducing agent DTT and incubating at 60 °C for 10 min. Then, 50 mM iodoacetamide (IAA) was added and incubated at room temperature (30 °C) for 30 min. The proteins in the supernatant were digested with trypsin enzyme at a ratio of 1:75 w/w (trypsin enzyme:protein sample) and incubated at 37 °C for 16 h. Ten percent trifluoroacetic acid (TFA) was added to the digestion mixture to adjust the pH to ≤ 3, and then the peptide mixture was purified using a C_18_ column.

### Peptide desalting

Peptide samples were passed through a C_18_ column GL-Tip TM SDB (GL Sciences Japan). The column was preconditioned by adding 100 μl of buffer B (0.1% TFA and 80% acetonitrile (ACN)) to a C_18_ column tip and equilibrated by adding 100 μl of buffer A (0.1% TFA and 5% ACN) to the C_18_ column tip. Then, the peptide samples were added to the C_18_ column tip and washed by adding 100 μl of buffer A. The desalted peptides were eluted by buffer B1 (0.1% TFA and 50% ACN), and the peptide eluents were dried by using a speed vacuum at 60 °C for 3 h. The dried peptide samples were dissolved in 5 μl of buffer containing 50% ACN, 0.1% formic acid (FA) and 20 μl 0.1% FA, and these peptide samples were desalted using 10 μl ZipTip columns (Millipore USA). The ZipTip columns were washed two times with 100% ACN and 50% ACN and three times with 0.1% FA. Then, the samples were loaded into ZipTip columns and washed with 0.1% FA. The peptide samples were finally eluted with buffer (40% ACN, 0.1% FA) and subsequently dried using speed vacuum.

### Proteome analysis by using liquid chromatography–tandem mass spectrometry (LC–MS/MS)

All peptide samples were subjected to quantitative proteome analysis by using an Agilent 1260 Infinity HPLC-chip/MS interfaced to the Agilent 6545 Q-TOF LC/MS system (Agilent Technologies, USA). ProtID-chip-150 II (Number G4240-62006) was used in the HPLC-chip/MS system. ProtID-chip-150 II contains a 40 nL trap column and a 75 μm × 150 mm separation column packed with Zorbax 300SB-C18 (5 μm). The mobile phase used for the capillary pump was a buffer containing 2% acetonitrile, 0.6% acetic acid and 2% FA in water at a flow rate of 0.4 μl/min, and those used for the nanopump were buffer A (0.6% acetic acid in water) and buffer B (0.6% FA in acetonitrile) at a flow rate of 0.4 μl/min with a linear gradient. Q-TOF MS/MS conditions were as follows: high resolution, 4 GHz; source temperature, 150 °C; capillary voltage, 1950 V; fragmentor voltage, 140 V; and flow rate, 6 L/min of drying gas. Positive ion mode and automatic data acquisition mode were used for all sample analyses. Automatic data acquisition was performed at a mass range of 100-140 m/z for MS mode and 80–2000 m/z for MS/MS mode. The acquisition rate was 3 spectra/sec for MS and automatic MS/MS mode.

According to the methods described by Kurdrid et al., the peptide samples were dissolved in 3% acetonitrile and 0.3% formic acid in water before analysis [11]. The peptide samples were loaded onto a 36-min gradient column, and the gradient was initiated at 5-15% buffer B for 0-2 min, increased to 15-35% for 2-30 min, increased to 35-60% for 30-32 min, maintained for 32-34 min and then reduced to 5% for 34-36 min. Column equilibration was performed in the negative mode run to clean up by using the polarity for 5 min. The peptide samples were analyzed by LC–MS/MS using MassHunter software (version B.06.01), with the following software settings: modification, carbamidomethylation (C), 600–6000 Da precursor MH+ and scan time range of 0–300 min. The MS/MS search used high stringency criteria; trypsin digestion, permitting up to 2 missed cleavages; carbamidomethylation (C) as a fixed modification; phosphorylation of Ser (S), Thr (T) and Tyr (Y) as variable modifications; precursor mass tolerance ±20 ppm; and product mass tolerance ±50 ppm. The reverse database scores used for % false discovery rate (% FDR) were calculated in search mode. Then, Spectrum Mill (version B.06.00.201HF1) was applied for protein identification by using a *Synechocystis* database. Principal component analysis (PCA) was used for analysis in the quality control mode of the MPP program. Statistical analysis was performed for the significance analysis, i.e., a t test against zero, a one-way ANOVA for each condition and a two-way ANOVA for two independent variables. The cutoff criterion was a p value less than or equal to 0.05. Then, the protein expression levels were detected using Mass Profiler Professional, or MPP (version 15.1), and statistical assessment was performed. The differentially expressed protein levels were detected using a significance cutoff criterion defined by the fold-change level. Specifically, proteins whose expression increased by a factor of at least 1.5 were considered upregulated proteins, and those whose expression decreased by a factor of at least 1.5 were considered downregulated proteins.

## Results

### Growth rate, chlorophyll *a*, and O_2_ evolution rate

*Synechocystis* sp. PCC6803 (WT) and mutant cells (MT or ∆Hik28) were grown for a period of 24 h in normal BG-11 (+NO_3_) and nitrogen depletion BG-11 medium (-NO_3_) under the optimal temperature, low- and high-temperature stress experiments. Low-temperature stress conditions were described in our previous research [11]. Under the combined stress, nitrogen depletion and high temperature, the growth rate, Chl *a* and O_2_ evolution rate decreased in both WT and ∆Hik28 cells. However, the Chl *a* and O_2_ evolution rate of ∆Hik28 cells were lower than those of WT cells (Table 1 and Suppl. Fig. 3).

**Table 1:** Cell density (OD_730_), chlorophyll a content and oxygen evolution rate of *Synechocystis* sp. PCC 6803 (WT) and ΔHik28 (MT) strains under high-temperature stress and nitrogen stress

Strain	BG-11 medium					
	+NO_3_			-NO_3_		
	0h	1h	24h	0h	1h	24h
Cell grown at 30^o^C						
*Cell density (OD*_*730*_*)*						
6803 (WT)	0.979+0.020	0.95+0.04	1.019+0.04	1.016+0.01	0.99+0.024	1.056+0.026
∆Hik28 (MT)	0.97+0.054	0.93+0.024	1.010+0.018	1.01+0.03	0.966+0.012	0.95+0.026
*Chlorophyll (mg/l)*						
6803 (WT)	9.49+1.18	10.22+2.02	12.43+1.63	10.23+0.26	10.97+1.99	11.53+2.54
∆Hik28 (MT)	10.35+0.79	10.15+1.10	10.92+0.60	11.13+0.41	10.75+1.07	11.13+0.67
*O*_*2*_ *evolution content*						
*(μmolO*_*2*_*mg*^*−1*^*Chl.hr*^*.− 1*^*)*						
6803 (WT)	413.73+32.69	398.66+12.24	354.10+20.97	411.73+31.15	393.33+17.55	321.6+32.56
∆Hik28 (MT)	363.86+25.75	410.93+23.49	286.4+29.79	359.2+29.10	318.66+31.66	239.2+24.40
Cell grown at 45^o^C						
*Cell density (OD*_*730*_*)*						
6803 (WT)	0.99+0.026	0.98+0.035	0.998+0.027	1.01+0.026	1.01+0.035	1.01+0.045
∆Hik28 (MT)	0.96+0.029	0.89+0.022	0.88+0.032	1.01+0.025	0.96+0.012	0.93+0.013
*Chlorophyll (mg/l)*						
6803(WT)	9.89+0.75	9.94+1.56	10.51+0.93	9.96+0.70	10.04+1.36	9.92+1.81
∆Hik28 (MT)	9.83+1.59	10.51+1.57	10.47+1.31	11.94+1.01	10.84+0.66	9.20+0.38
*O*_*2*_ *evolution content*						
*(μmolO*_*2*_*mg*^*−1*^*Chl.hr.-* ^*1*^*)*						
6803 (WT)	421.86+19.66	394.93+18.63	209.86+14.51	418.00+12.85	386.93+17.89	177.06+11.71
∆Hik28 (MT)	356.66+34.67	331.2+13.36	173.06+12.11	350.53+31.64	278.13+17.25	142.13+8.92

### Protein–protein interaction by yeast two-hybrid system

In yeast two-hybrid experiments, the interaction of SPLC1_S041070 (Hik28) with SPLC1_S630120 (GlnA) and SPLC1_S240970 (GlnB) yielded positive results consisting of blue colonies on SD medium lacking Ade, His, Leu, Trp, X-α-gal and AbA dropout (QDO/X/A) (Suppl. Fig. 2). However, the interaction of Hik28 with GlsF has been reported previously [11].

### Quantitative proteome analysis and expression patterns of differentially expressed proteins

**Fig. 1:**
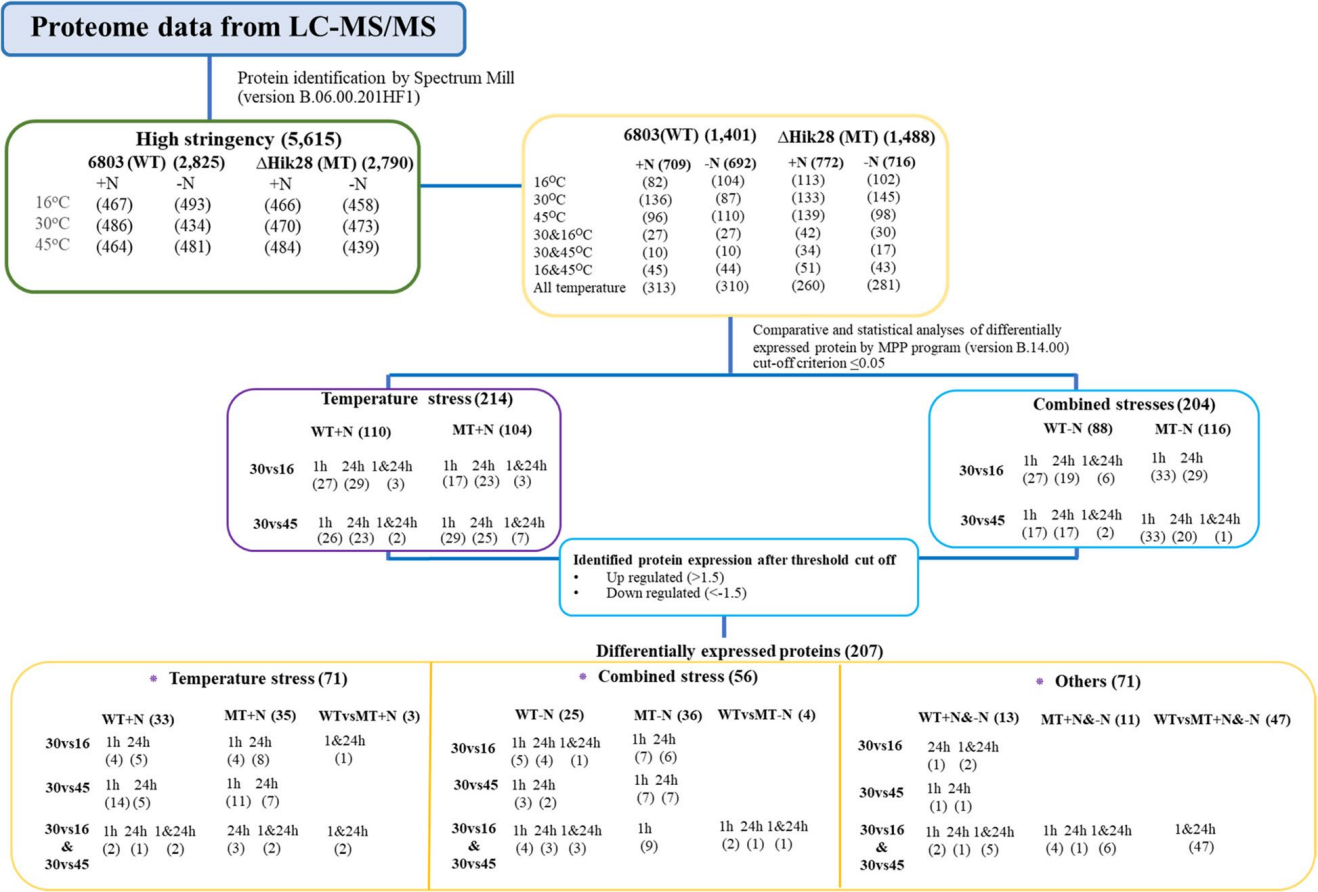
Workflow for proteome data analysis. The number in parentheses shows the proteins of the *Synechocystis* sp. PCC 6803 (WT) and ∆Hik28 (MT) strains obtained from the proteomic analysis under the experimental conditions: temperature stress (non-optimal temperature in the presence of nitrogen (+N)) and combined stress (non-optimal temperature and nitrogen depletion (-N))

**Fig. 2:**
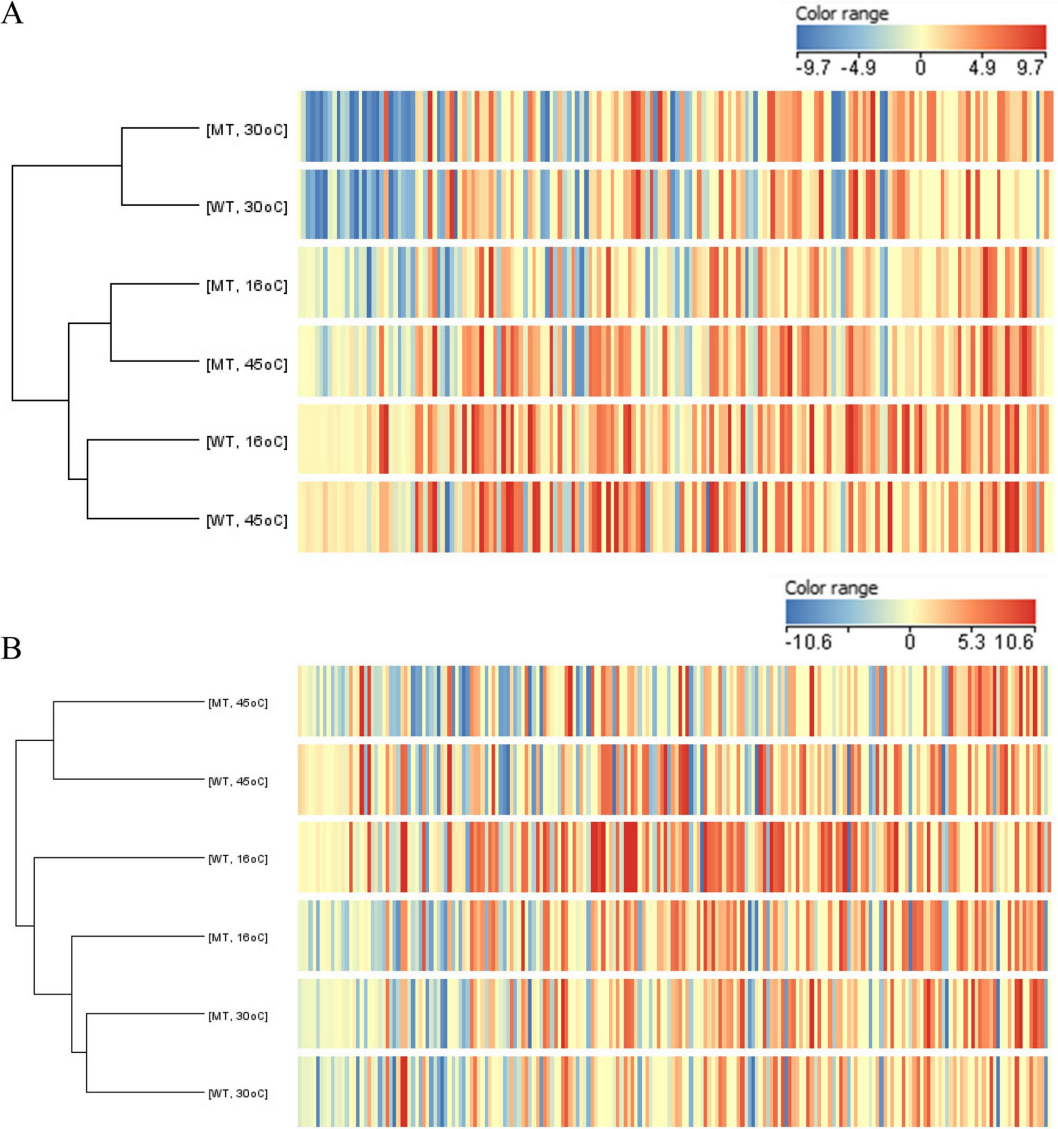
Heatmap of the differentially expressed proteins found in the WT and MT strains after (**A**) 1 h and (**B**) 24 h of the stress exposure, clustered by MPP program version 15.1

The proteomes of *Synechocystis sp.* PCC6803, WT and MT cells grown in normal BG-11 and nitrogen-free BG-11 medium under optimal temperature and after exposure to low (16 °C) temperature stress and high (45 °C) temperature stress for 0, 1, and 24 h were quantitatively analyzed by LC–MS/MS. In total, 5,615 proteins were obtained from the high-stringency MS/MS search, comprising 2,825 proteins from the WT strain and 2,790 proteins from the MT strain (Fig. 1). After clustering of the differentially expressed proteins by using hierarchical clustering of MPP program version 15.1 (Fig. 2), it showed that the group of immediate response proteins, which detected 1 h after exposure to the combined stress, found in the two strains, WT and MT, under the same growth temperature were related. However, the group of delay response proteins, which expressed 24 h after the stress exposure, of MT grown at 16^o^C was in the same hierarchy as that of the WT and MT grown at 30^o^C. The absence of Hik28 led to the change in cell response to the combined stress of low temperature and nitrogen, and the expression pattern was similar to that of under the optimal condition of both strains.

When the number of differentially expressed proteins was considered, under optimal-, low- and high-temperature conditions, respectively, 486, 467 and 464 proteins were found in WT, whereas 470, 466 and 484 proteins were found in MT (Suppl. Table 3 and Suppl. Table 4). Moreover, 434, 493 and 481 proteins in WT and 473, 458 and 439 in MT∆Hik28 were identified under combined temperature and nitrogen stress (Suppl. Table 5 and Suppl. Table 6). Comparative analysis of the differentially expressed proteins in both WT and MT was performed by the MPP program with a cutoff *p* value of <0.05. A total of 214 and 204 proteins were found to be differentially regulated under temperature and combined stress, respectively, in both strains. A total of 207 differentially expressed proteins under all experimental conditions were identified by cutoff criteria of fold change ≤ -1.5 and ≥1.5 (Fig. 1 and Suppl. Table 7A). Under temperature stress, 33, 35 and 3 proteins were differentially expressed in WT, MT and both strains, respectively (Suppl., Fig. 4, Suppl. Table 7B, 7E and 7H), whereas 25, 36 and 4 proteins were found under combined temperature stress and nitrogen-depletion stress (Suppl. Fig. 4, Suppl. Table 7C, 7F and 7I). Moreover, the expression levels of 13, 11 and 47 proteins were detected in WT, MT and both strains under more than one experimental condition; these proteins are designated as “others” in Fig. 1 and Suppl. Fig. 4, and the details of the proteins are shown in Suppl. Table 7D, 7G and 7J.

**Fig. 3:**
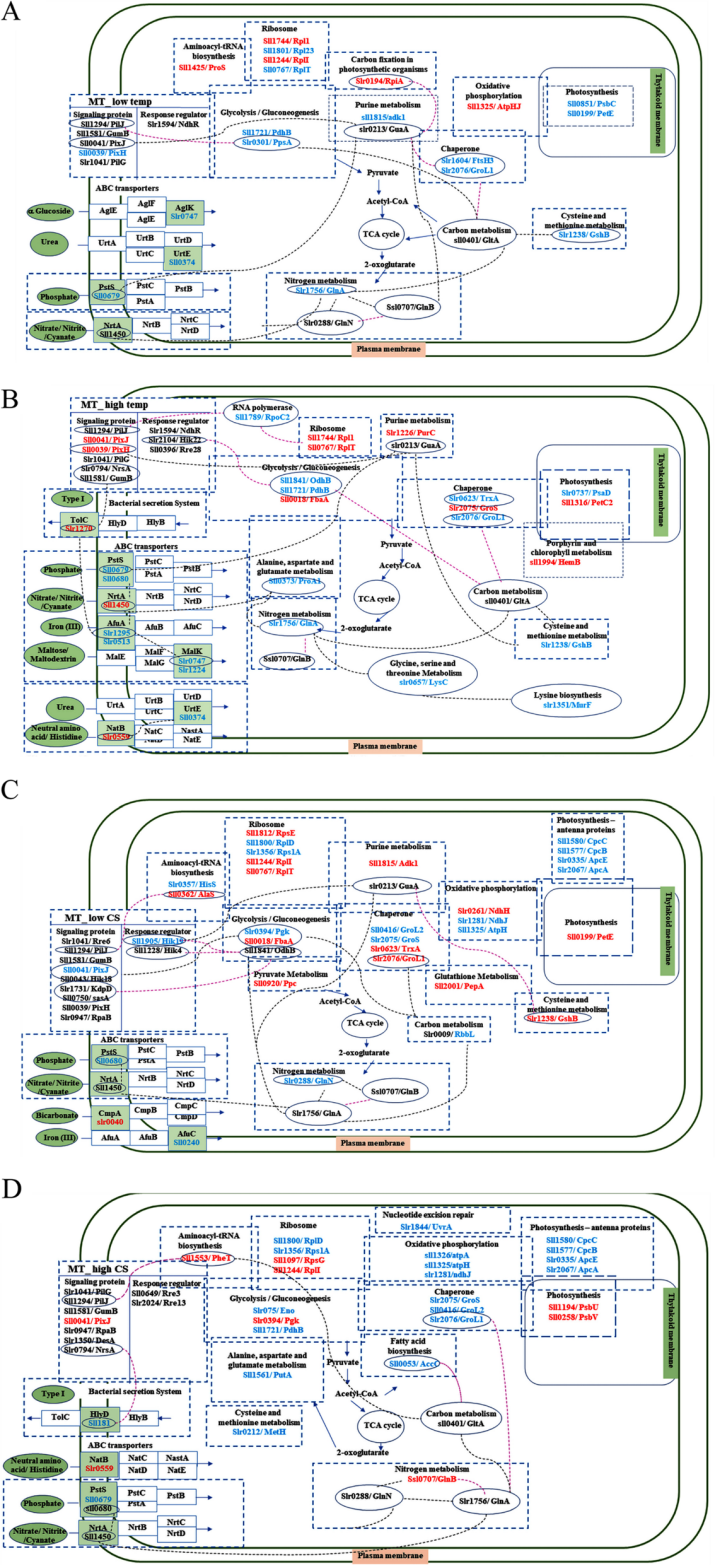
The protein–protein interaction (PPI) network of the two-component system, their response regulators and the up- and downregulated proteins found in the *Synechocystis* ∆Hik28 (MT) strain under **A** low-temperature stress, **B** high-temperature stress, **C** combined low-temperature and nitrogen-depletion stress and **D** combined high-temperature and nitrogen-depletion stress. The PPI network of the regulated proteins in metabolic pathways is illustrated in the boxes bounded by the dotted lines. The up- and downregulated proteins are shown in red and blue letters, respectively

Since the signaling proteins and response regulators are regulated by posttranslational modification rather than the expression level [11], the proteomic data before the differential expression analysis in terms of fold change were also considered in this case. In the WT strain, under low-temperature stress in the presence of nitrogen, the two-component systems involved in quorum and osmolarity sensing were uniquely detected, whereas under high-temperature conditions, the efflux system protein involved in nickel and cobalt tolerance was found (Suppl. Fig. 5A-B). Under combined nitrogen and temperature stress, in addition to the two-component system involved in nitrogen metabolism and chemotaxis, metal-sensitive and osmolarity-sensing proteins were uniquely found after low- and high-temperature exposure, respectively (Suppl. Fig. 5C-D). In the MT, in which Hik28 was absent, a different set of TCSs was found under the combined stress. Polysaccharide biosynthesis/export and twitching motility proteins (Fig. 3A-D). Moreover, in the case of combined low-temperature and nitrogen-depletion stress, Sll5060, Sll1228 and Sll1367 were detected in the Hik28-deletion strain, whereas under the combination of high-temperature and nitrogen-depletion stress, the proteins involved in cation efflux, chemotaxis (CheY) and osmolarity sensing were detected together with nitrogen assimilation proteins and the fatty acid–metabolizing enzyme delta12-desaturase (Fig. 3C-D).

## Discussion

In the present study, the effect of combined temperature and nitrogen stress on the cell growth of WT and MT strains was studied by measuring cell density, Chl *a* content and O_2_ evolution. When the ΔO_2_ evolution of the cells grown in the presence and absence of nitrogen was calculated (Table 1 and Suppl. Fig. 3), the higher value of ΔO_2_ evolution found in the MT cells indicated poorer adaptation to N stress. This result implies that Hik28 most likely plays a role in cell growth via the photosynthetic mechanism in response to N stress.

Moreover, the clustering results, shown in Fig. 2B, of the differentially expressed proteins obtained from comparative proteomic analysis of the WT and MT strains supported the critical role of Hik28 in response to the temperature downshift due to the absence of Hik28 led to similar protein expression pattern of MT under 16^o^C and that of the WT and MT strains under the optimal temperature. Sensor histidine kinases in two-component signal transduction systems, including Hik28, enable cyanobacteria to sense, respond, and adapt to environmental changes, stressors, and growth conditions. It is well known that in the response mechanism, the phosphoryl group is transferred from the autophosphorylated sensor histidine kinase to a response regulator (RR), which subsequently affects cellular physiology by regulating gene expression [14]. After signal retrieval, the activated RR binds to its target promoter regions and subsequently regulates the transcriptional machinery [15]. Moreover, in the stress response mechanism, the two-component system and ABC transporters are functionally related in the transportation of substrates, including peptides, amino acids, sugars and antibiotics [16–19].

### Effects of Hik28 deletion

#### Two-component signal transduction system and ABC transporter:

Based on the proteomic data, a unique set of signaling and response regulator proteins were detected in the WT and mutant strains under temperature and combined stress (Fig. 3A-D and Suppl. Fig. 5A-D). The data clearly indicated the effect of Hik28 deletion at the level of abiotic stress sensing and the specific set of response regulators involved. Furthermore, a two-component system is known to tightly regulate ABC transporters, which are an important class of proteins that transport various extracellular substrates, including peptides, amino acids, sugars and antibiotics [20]. After the two-component system senses and transfers the signal from the environmental stress, proteins in the ABC transporter group are one of the immediate responses of the cells to the stress that is regulated by the TCS. The TCS induces a quick and specific response to stimuli, and both the TCS and the ABC transporter system have demonstrated their ability to sense biotic and abiotic stress and substrates, including peptides, amino acids, sugars and antibiotics; however, the exact mechanism is not fully established.

**Fig. 4:**
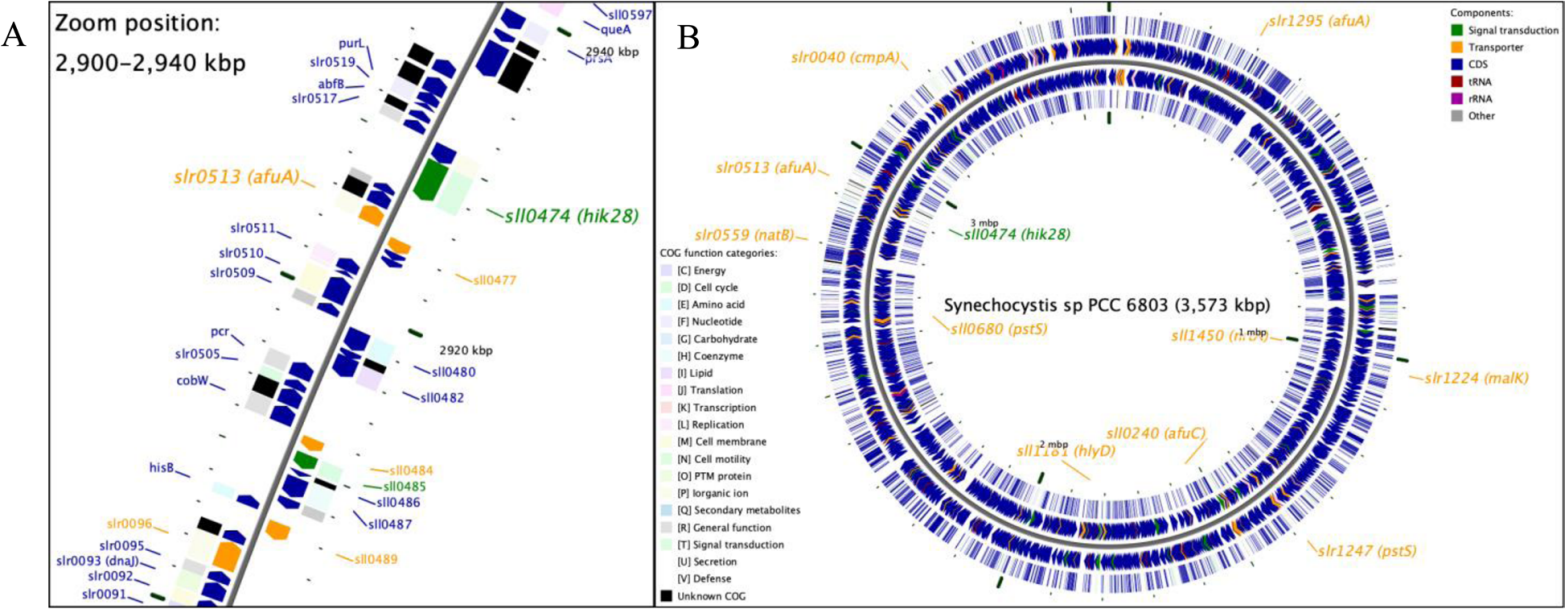
An illustration of the loci on *Synechocystis* sp. PCC6803 genome using CGview and genome feature information of the encoded TCS genes and the ABC transporters found in the two strains, WT and MT, under each experimental condition **A** zoom-in of the region surrounding Hik28-encoded gene and **B** *Synechocystis* sp. PCC6803 genome with Hik28-and ABC transporter- genes labeled

In *Synechocystis sp.* PCC6803, WT strain, there are a total of 73 ABC transporter proteins, and 15 of them were detected in the proteomic data. It has been reported that the genes encoding TCS proteins and the ABC transporters that they regulate are located close together in the genome [21, 22]. Therefore, the loci of the encoded TCS genes and the ABC transporters found in the two strains under each experimental condition are shown in Fig. 4. An illustration of the loci using CGview and genome feature information showed that the iron transporter, Slr0513: FutA2 or AfuA, was in a position upstream of Hik28 and that the protein was downregulated in the Hik28-deletion mutant in response to elevated temperature, whereas it was upregulated in the WT under the combined stress of high temperature and nitrogen depletion. *Synechocystis sp.* PCC6803 was reported earlier to have a 10-fold higher demand for iron than *Escherichia coli* to sustain photosynthesis. Thus, (i) the evidence supported the necessity of the iron-binding protein Afu/FutA2 for growth (Badaruh et al. 2008) in the WT, and the data showed that the regulation of this transporter was Hik28-dependent; (ii) the evidence showed that the genes coding for the TCS proteins, e.g., Hik28, might regulate ABC transporters, e.g., Afu/FutA2, whose genes are nearby in the genome.

In addition to the iron transporter, the group of ABC transporters, whose regulation can be considered Hik28 dependent, were urea and α-glucoside transporters. These two transporters were downregulated in the Hik28-deletion mutant and had protein–protein interactions with proteins involved in the metabolic process to concentrate carbon dioxide. The two transporters were differentially expressed in response to low-temperature stress, supporting the finding that Hik28 plays a critical role as a signaling molecule in the low-temperature response mechanism [11].

Furthermore, it is noteworthy that the hypothetical and unknown proteins located upstream (Slr0516) and downstream (Sll0493) of Hik28 were found to have protein–protein interactions with Hik28 according to STRING [23]. In addition to Hik28, Slr0516 also interacts with biopolymer transporters, whereas Sll0473 interacts with nitrate and bicarbonate transporters (Suppl. Fig. 6A). Moreover, Slr0517, located downstream of Hik28, was functionally related to Hik28 and proteins in glutamine metabolic processes and purine biosynthesis in the PPI network (Suppl. Fig. 6A). In accordance with previous reports, the TCS and the ABC transporter located adjacent to it in the genome were related, thus supporting the finding that the TCS regulates nearby ABC transporter genes [21, 22].

The ABC transporters of the two strains, WT and MT, were compared under combined stress; in the mutant, which lacked Hik28, the ABC transporters responsible for molybdate/sulfate, xenobiotic, and phosphate transporter were revealed to be downregulated proteins (Fig. 3C-D and Suppl. Table 7). However, the deletion of Hik28 combined with temperature stress has negative effects on the iron, osmolyte and sugar transfer systems, whereas the urea transporter Sll0374: UrtE was downregulated after high-temperature exposure and vice versa under low-temperature stress. Interestingly, Slr0559, an ABC transporter for general L-amino acids, was upregulated in the mutant strain under high-temperature stress regardless of the nitrogen supply. However, in the WT under the three experimental conditions, low temperature, combined low-temperature and nitrogen stress, and combined high-temperature and nitrogen stress, and in the MT under the condition of combined low-temperature and nitrogen stress specifically, the proteomic data showed upregulation of the phosphate transporter Slr1247: PstS, suggesting the necessity of phosphate for cyanobacterial growth. Indeed, phosphate is a key growth limiting nutrient, particularly in freshwater cyanobacteria [24].

Other proteins in the class of ABC transporters involved in the control of the C/N ratio inside the cells are Slr0040: bicarbonate transporter and Sll1450: nitrate/nitrite/cyanate transporter. The nitrate/nitrite/cyanate transporter was differentially expressed only in the absence of Hik28 in response to high-temperature stress and in combination with nitrogen depletion. Moreover, the PPI network of these transporters showed interactions with proteins involved in nitrogen metabolism and iron and bicarbonate transport systems (Suppl. Fig. 6F). The bicarbonate transporter was downregulated under the combined stress of nitrogen depletion and low temperature in the Hik28-deletion mutant, whereas the protein expression level in the WT was decreased only under low-temperature stress, showing its Hik28-independent regulation. (Fig. 3A-D). The results suggested that *(i)* Hik28 possibly played a role in nitrogen assimilation and *(ii)* the bicarbonate requirement of the cells was reduced in response to low-temperature conditions. The evidence obtained from the proteome analysis supported the importance of the regulation of the C/N ratio in the survival and growth of cyanobacteria [11], especially under stress conditions. Furthermore, the proteins in the bacterial secretion system, HlyD and TolC, were differentially expressed under high temperature in the Hik28-deletion strain (Fig. 3B). The results indicated that the absence of Hik28 and exposure to the combined stress had direct effects on the group of ABC transporters that transfer nutrients across the periplasmic membrane.

#### Response of metabolic pathways to combined stress

The metabolic pathways affected by the combined stress of immediate temperature shift and nitrogen depletion were comparatively analyzed by strain and by growth condition, as shown in Fig. 3A-D and Suppl. Fig. 5A-D. Taken together, the proteomic data on differentially expressed proteins and the protein–protein interaction network demonstrated changes in the expression levels of N metabolism proteins, GlnA, GlnB and GlnN under temperature stress and combined stress in the absence of Hik28 (Suppl. Fig. 6B-I), supporting the report by Kurdrid et al. that Hik28 is critically involved in N metabolism. Moreover, the results at the proteome level showed the effect on fatty acid biosynthesis in mutant cells in response to the combined stress of high temperature and nitrogen depletion, which was in accord with the fatty acid biosynthesis data showing the drastic accumulation of C16:1^Δ9^ reported by Kurdrid et al. (2020). It is also noteworthy that the proteins involved in oxidative phosphorylation were significantly upregulated in the MT strain in response to temperature downshift and its combination with nitrogen stress. The upregulation of F-type H+-transporting ATPase and subunit a of ATP synthase suggested that the mutant cells had increased energy requirements under stress; therefore, the absence of Hik28 led to the requirement of ATP under low-temperature and combined stress (Suppl. Fig. 6B and D), which strongly supported the evidence that Hik28 played a crucial role in the low-temperature stress response mechanism [11].

According to a report by Kurdrid et al., oxygen evolution and chlorophyll a content decreased in mutant cells after a temperature shift to 16 °C in the presence of nitrogen, showing the negative effect of Hik28 deletion on the photosynthetic apparatus [11]. In the present study, the upregulation of proteins in PSI, PSII, the cytochrome *b6f* complex and photosynthetic electron transport was observed in the WT strain (Suppl. Fig. 6F). However, in the MT, under the combined stress of low temperature and N depletion, PetE, a protein in the photosynthetic electron transport system, was upregulated (Suppl. Fig. 6D), whereas it was downregulated after a temperature downshift (Suppl. Fig. 6B), supporting the evidence found by Kurdrid et al. [11]. In response to a temperature upshift or its combination with N stress, the proteins in PSII and the cytochrome *b6f* complex were upregulated. The comparative proteome data indicated that Hik28 may have an effect on PetE under low-temperature stress and in combination with N stress; however, the expression of other proteins in the photosynthetic system was independent of Hik28. Moreover, the expression level of ribose-5-phosphate isomerase, RpiA, during carbon fixation by this photosynthetic organism increased only in the MT strain after a temperature shift from 35 °C to 16 °C, suggesting that the mutant cells under low-temperature stress acquired higher levels of carbon than WT cells (Fig. 3A, Suppl. Fig. 6B and F). This evidence supported the finding by Hutchings et al. that RpiA, which plays a key role in the pentose phosphate pathway, is directly connected to fatty acid biosynthesis, which is regulated under temperature stress, by NADP/NADPH metabolism [25].

Another pathway involved in photosynthesis is porphyrin metabolism, in which the photosynthetic pigment chlorophyll a is synthesized. The enzymes involved in porphyrin metabolism, such as porphobilinogen synthase (HemE), were upregulated at elevated growth temperatures in the absence of Hik28 protein (Fig. 3B), whereas the expression level of protochlorophyllide oxidoreductase (Por) decreased after a temperature upshift in the WT strain (Suppl. Fig. 5B). The results showed that the proteins involved in porphyrin metabolism were regulated in response to elevated growth temperature and that Hik28 deletion caused differences in the regulation of porphyrin metabolism. Moreover, regardless of Hik28 deletion and stress conditions, the ribosomal proteins and the proteins in the oxidative phosphorylation pathway were upregulated, suggesting a need for protein biosynthesis and energy for the stress response mechanism and other metabolic processes.

## Conclusion

Comparative proteomic analyses of *Synechocystis sp.* PCC6803 and its Hik28-deletion mutant under temperature stress and a combination of temperature and nitrogen-depletion stress revealed the group of response regulators, e.g., cation efflux, chemotaxis and osmolarity sensing, that are involved with the TCS signal protein Hik28. After the cells sensed and transferred the signal, the proteins in the group of transporters were differentially regulated to manage the movement of nutrients across the periplasmic membrane. The Hik28-dependent ABC transporters were iron, urea and α-glucoside transporters, whereas the Hik28-independent ones were phosphate and bicarbonate transporters. By combining the proteome data and the PPI information, we were able to illustrate the network structure of the group of proteins and metabolisms/pathways that were affected by the mutation under temperature stress and combined temperature and nitrogen stress (Fig. 3 and Suppl. Fig. 5). The metabolic processes affected by the absence of Hik28 and the designated stress conditions were the nitrogen and carbon metabolic processes that regulate the C/N ratio, the photosynthetic apparatus and fatty acid biosynthesis.

## Supplementary Information

**Additional file 1.**

**Additional file 2.**

**Additional file 3.**

**Additional file 4.**

**Additional file 5.**

**Additional file 6.**

**Additional file 7.**

**Additional file 8.**

**Additional file 9.**

**Additional file 10.**

**Additional file 11.**

**Additional file 12.**

**Additional file 13.**

## Data Availability

• The datasets generated and/or analyzed during the current study are available in this published article, supplementary information files and in our data repository, http://www.cyanopro.net/dl/proteome2022feb-syencho-hik28/ with username: reviewer, and password: wanna2C. • The mass spectrometry proteomics data have been deposited to the ProteomeXchange Consortium via the PRIDE partner repository with the dataset identifier PXD032795. Submission details: **Project Name:** Synechocystis-Hik28 stress response **Project accession:** PXD032795 **Project DOI:** Not applicable Reviewer account details: **Username:** reviewer_pxd032795@ebi.ac.uk **Password:** ZyZbr1X1
