# Supplementary material for: Hik28-dependent and Hik28-independent ABC transporters were revealed by proteome-wide analysis of ΔHik28 under combined stress
Source: BMC Mol Cell Biol. 2022 Jul 6;23:27. doi: 10.1186/s12860-022-00421-w (PMC9258054; doi:10.1186/s12860-022-00421-w)
Supplement: Supplementary file 1 — Additional file 1. [file 12860_2022_421_MOESM1_ESM.docx]

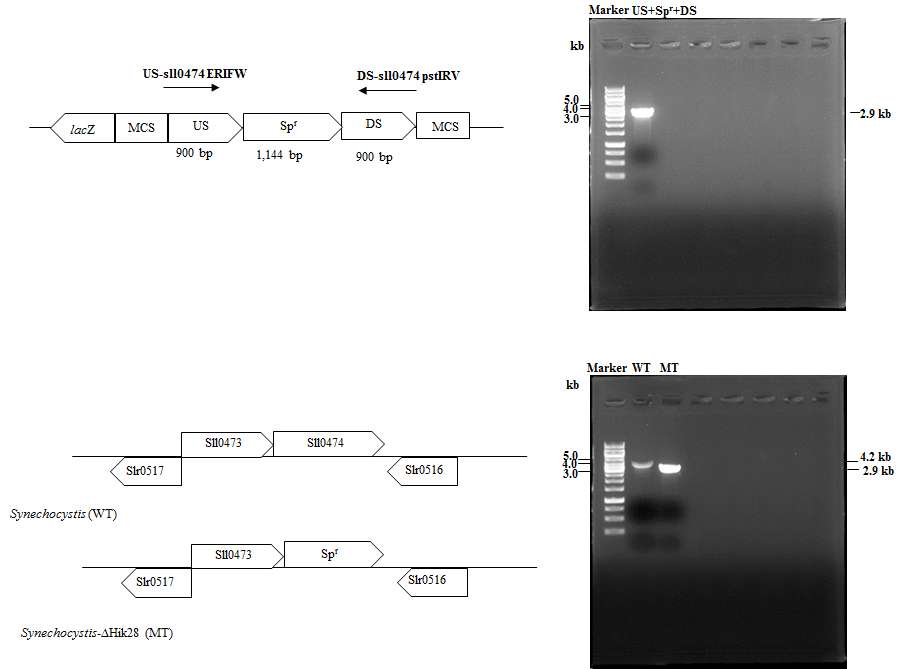


A

B

C

D

**Supplementary Figure 1** Construction of the *Synechocystis*-∆Hik28 (MT) strain; (A) upstream (US) and downstream (DS) of *hik*28 gene and spectinomycin resistant gene (Sp^r^) were inserted into pGEMT-easy plasmid, (B) the PCR product of US, Sp^r^ gene and DS fragments was amplified by using US-sll0474ERIFW and DS-sll0474 pstIRV primers, (C) the *hik28* was replaced by spectinomycin resistant (Sp^r^), and (D) the PCR product of *hik*28 gene in *Synechocystis* (WT) and Sp^r^ gene in ∆Hik28 (MT) strain were amplified by using US-sll0474ERIFW and DS-sll0474 pstIRV primers (modified from Kurdrid, 2020).
