# Supplementary material for: Hik28-dependent and Hik28-independent ABC transporters were revealed by proteome-wide analysis of ΔHik28 under combined stress
Source: BMC Mol Cell Biol. 2022 Jul 6;23:27. doi: 10.1186/s12860-022-00421-w (PMC9258054; doi:10.1186/s12860-022-00421-w)
Supplement: Supplementary file 2 — Additional file 2. [file 12860_2022_421_MOESM2_ESM.docx]

**Supplementary Table1**. Oligonucleotide primers used in the construction of ∆sll0474 gene

| Primer | Sequence (5’-3’) |  |
| --- | --- | --- |
| Construction of deletion | |  |
| Upstream of sll0474 |  |  |
| US-sll0474 ERIFW | CGGAATTCCACAGCCGCTCATAGTAGG |  |
| US-sll0474 HdIIIRV | CCCAAGCTTTCTTAAGACCGCTCCACA |  |
| Downstream of sll0474 |  |  |
| DS-sll0474 KpnIFW | TTGGTACCCGACGAGAACCATCGGGCA |  |
| DS-sll0474 pstIRV | GGGCTGCAGAAATTGAGCCTGAATCGGTTC |  |
| Spectinomycin resistant gene | |  |
| Spr-HIndIIFW | CCCAACCTTATTTGCCGACTACCTTGGT |  |
| Spr-KpnIRV | CGGAATTCGTCATGTCCTCAGCATTTGC |  |
