# Supplementary material for: Hik28-dependent and Hik28-independent ABC transporters were revealed by proteome-wide analysis of ΔHik28 under combined stress
Source: BMC Mol Cell Biol. 2022 Jul 6;23:27. doi: 10.1186/s12860-022-00421-w (PMC9258054; doi:10.1186/s12860-022-00421-w)
Supplement: Supplementary file 3 — Additional file 3. [file 12860_2022_421_MOESM3_ESM.docx]

**Supplementary Table 2** Oligonucleotide primers used for the gene construction in yeast two hybrid system

| Primer | Sequence (5’-3’) |
| --- | --- |
| SPLC1_S041070 | Multi-sensor Hybrid Histidine Kinase (Hik28) |
| SPLCHik28_FW | CGG AAT TCA TGG CAA GCA ACT CAT CA |
| SPLCHik28_RV | GGG CTG CAG TTA TAA GTC TAA AAT TTC CTG |
| SPLC1_S630120 | Glutamine synthetase (GlnA) |
| SPLCglnA_FW | TTT TCC ATG GAT GCC CAC GAC AGC CCA A |
| SPLCglnA_RV | TTT GGA TCC TTA GCA ATC GTA GTA GAG AGA |
| SPLC1_S240970 | Nitrogen regulatory protein P-II (GlnB) |
| SPLCglnB_FW | GGA ATT CGT GAA AAC CTT GAA AAA GA |
| SPLCglnB_RV | TGG GAT CCT TAA ATT GCT TCT AGG T |
