# Supplementary material for: Hik28-dependent and Hik28-independent ABC transporters were revealed by proteome-wide analysis of ΔHik28 under combined stress
Source: BMC Mol Cell Biol. 2022 Jul 6;23:27. doi: 10.1186/s12860-022-00421-w (PMC9258054; doi:10.1186/s12860-022-00421-w)
Supplement: Supplementary file 4 — Additional file 4. [file 12860_2022_421_MOESM4_ESM.docx]

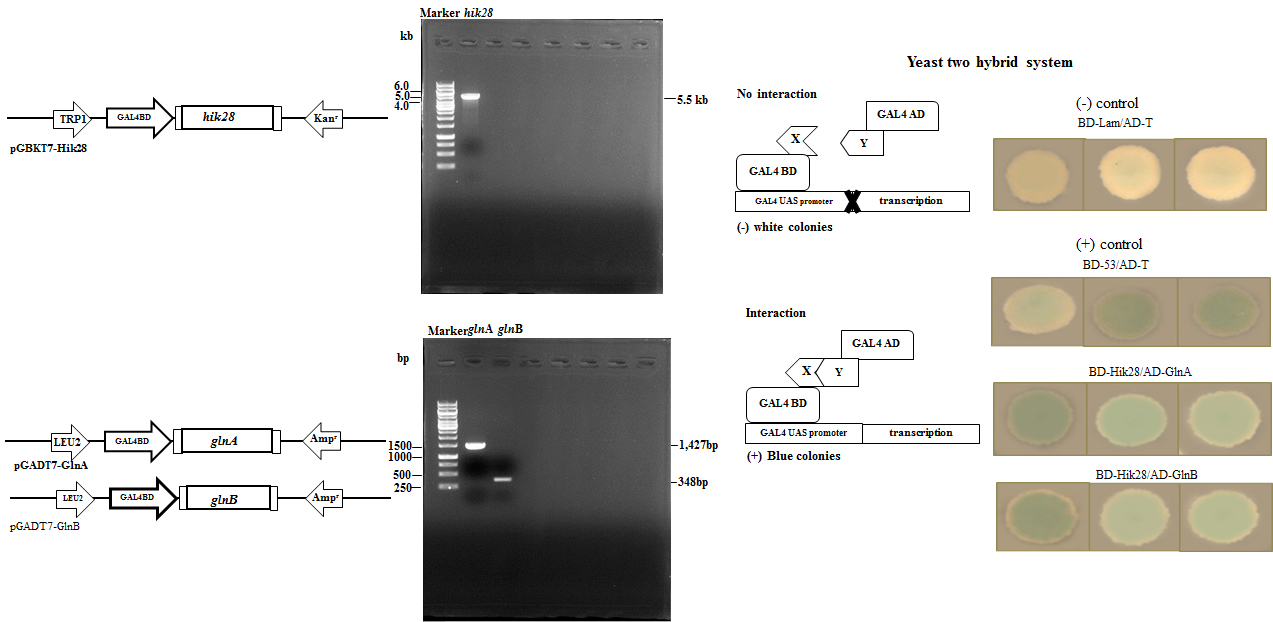


C

A

B

**Supplementary Figure 2** Yeast two hybrid system; (A) the construction of *hik*28 gene in pGBKT7*,* (B) *gln*A and *gln*B genes in pGADT7 vector, (C) the negative result of AD-T and BD-Lam (white colonies) and the positive results (blue colonies) of AD-T and BD-53, *Arthrospira-*Hik28 and its client proteins, GlnA and GlnB, (modified from Kurdrid, 2020).
