# Supplementary material for: Hik28-dependent and Hik28-independent ABC transporters were revealed by proteome-wide analysis of ΔHik28 under combined stress
Source: BMC Mol Cell Biol. 2022 Jul 6;23:27. doi: 10.1186/s12860-022-00421-w (PMC9258054; doi:10.1186/s12860-022-00421-w)
Supplement: Supplementary file 5 — Additional file 5. [file 12860_2022_421_MOESM5_ESM.docx]

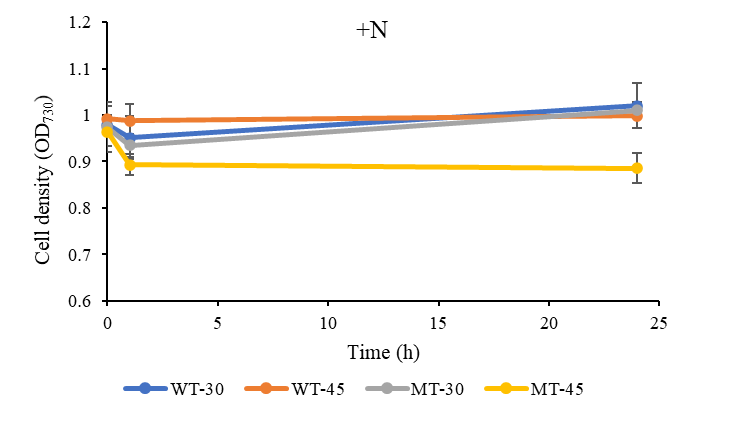

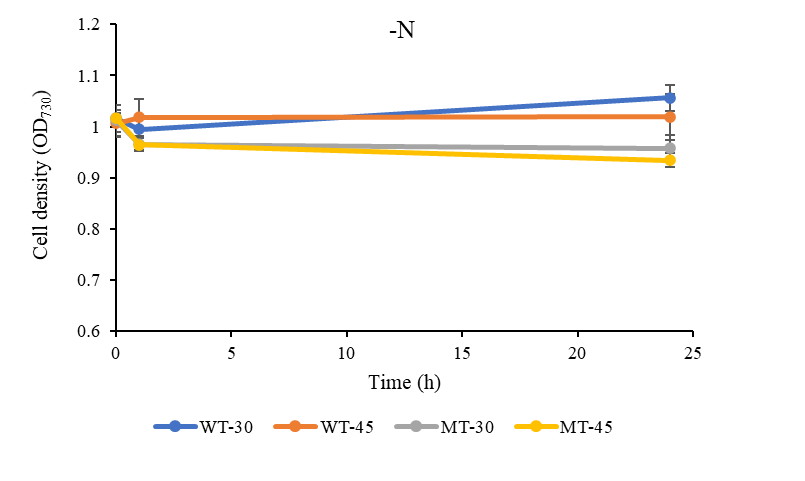

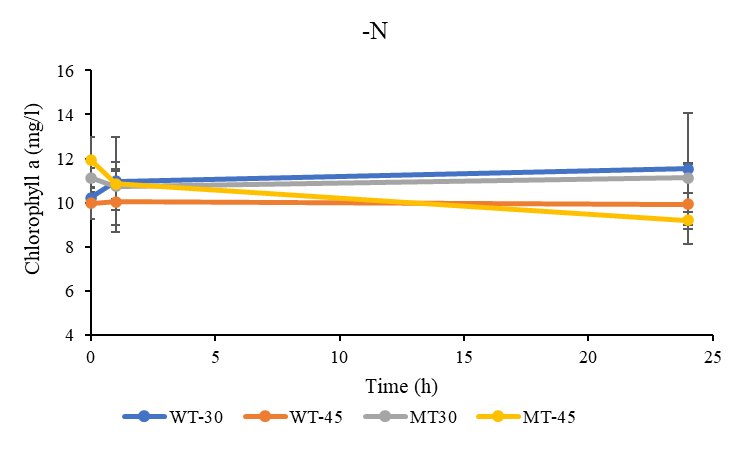

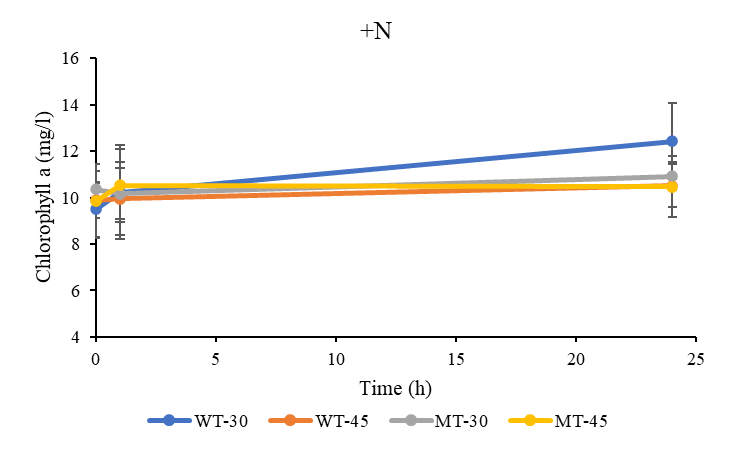

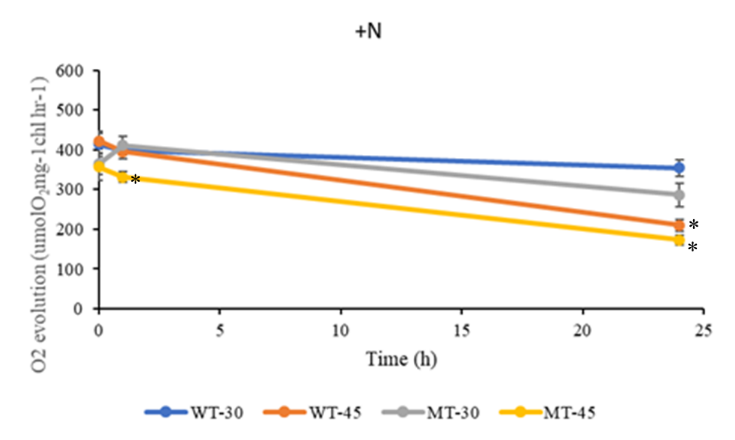

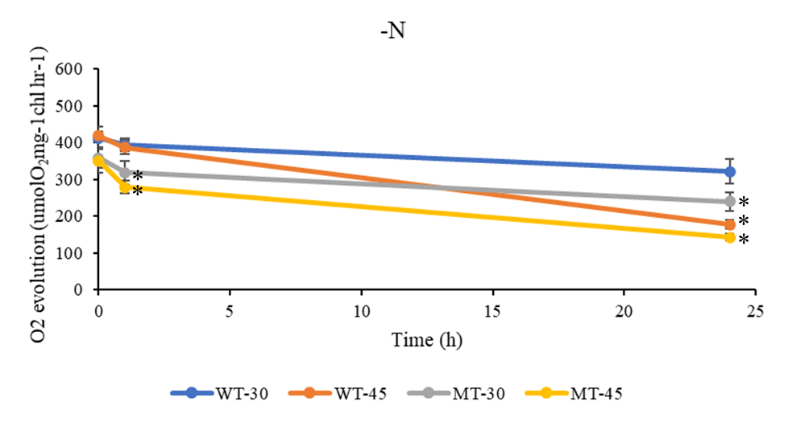


**Supplement Figure 3** Cell density (OD_730_), Chl *a* content and O_2_ evolving activity of *Synechocystis*-WT and MT strains grown in BG-11 (+N) and BG-11 medium nitrogen depletion (-N) under the optimal temperature (30^o^C) and high temperature stress (45^o^C) were measured before the stress exposure, 0 h, and 1 and 24 h after the stress exposure. The values represent the mean ± SD of three independent experiments (significance level * at p < 0.05).
