# Supplementary material for: Hik28-dependent and Hik28-independent ABC transporters were revealed by proteome-wide analysis of ΔHik28 under combined stress
Source: BMC Mol Cell Biol. 2022 Jul 6;23:27. doi: 10.1186/s12860-022-00421-w (PMC9258054; doi:10.1186/s12860-022-00421-w)
Supplement: Supplementary file 11 — Additional file 11. [file 12860_2022_421_MOESM11_ESM.docx]

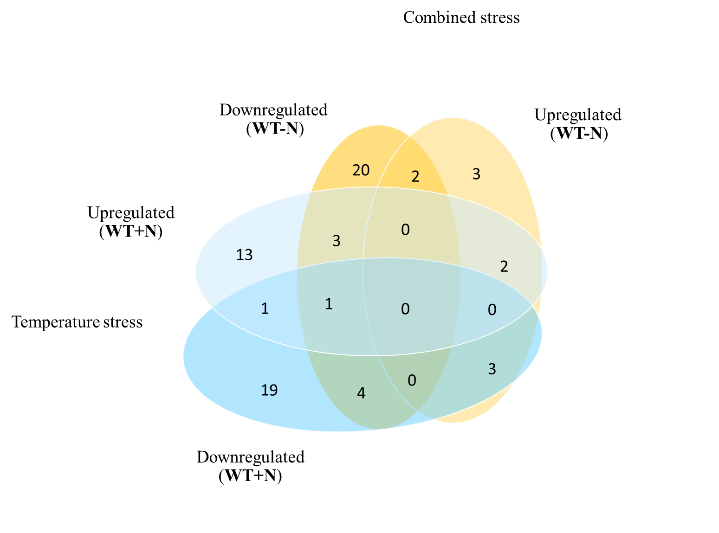


A


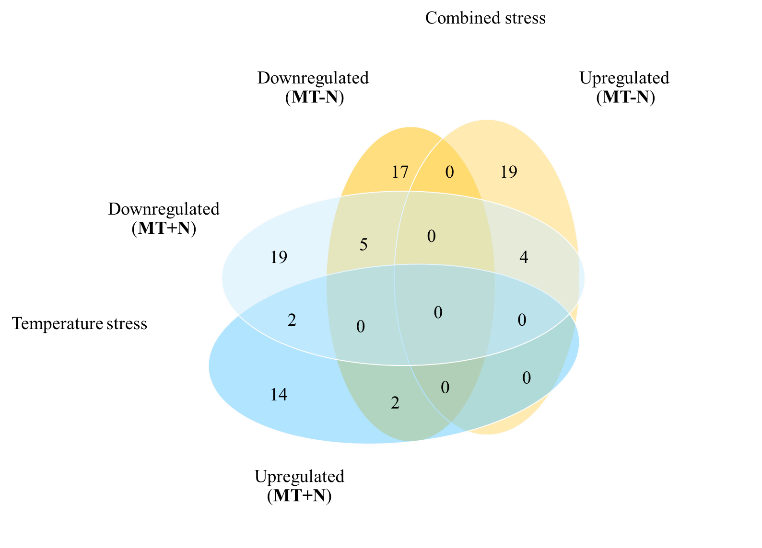


B


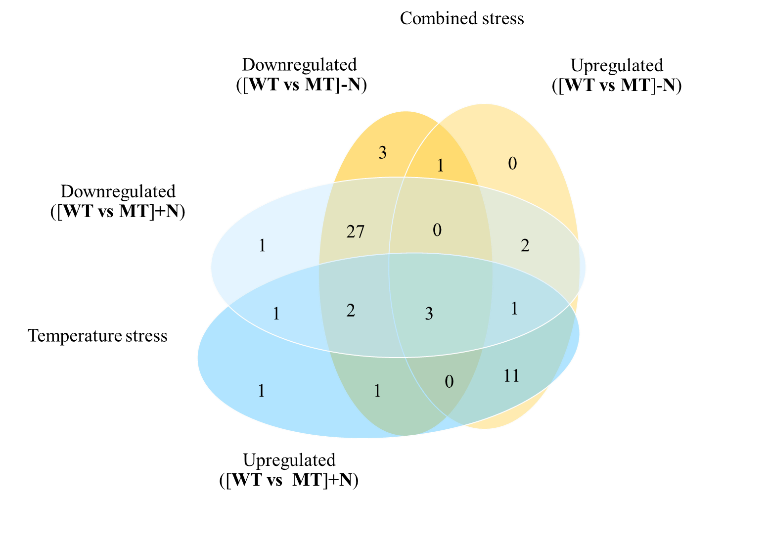


C

**Supplementary Figure 4** Venn diagram showed the numbers of up- and downregulated proteins of (A) *Synechocystis*-WT, (B) MT and (C) both of WT and MT strains under temperature stress, combined stress and others.
