# Supplementary material for: Hik28-dependent and Hik28-independent ABC transporters were revealed by proteome-wide analysis of ΔHik28 under combined stress
Source: BMC Mol Cell Biol. 2022 Jul 6;23:27. doi: 10.1186/s12860-022-00421-w (PMC9258054; doi:10.1186/s12860-022-00421-w)
Supplement: Supplementary file 12 — Additional file 12. [file 12860_2022_421_MOESM12_ESM.docx]

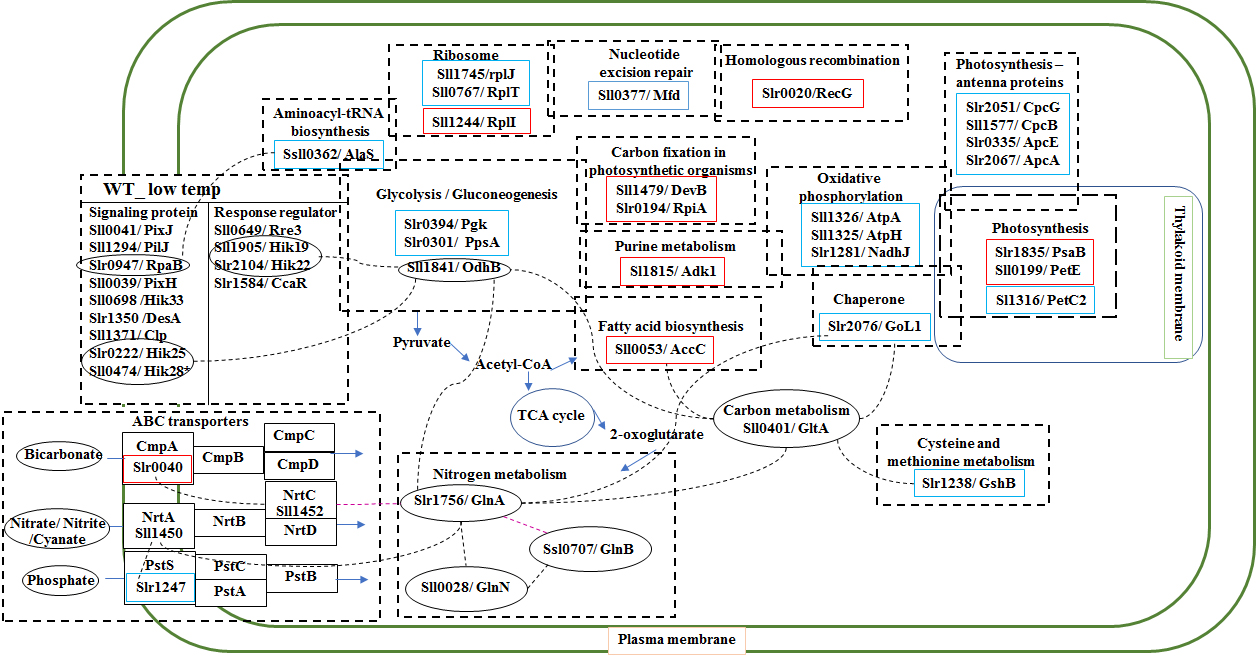


A


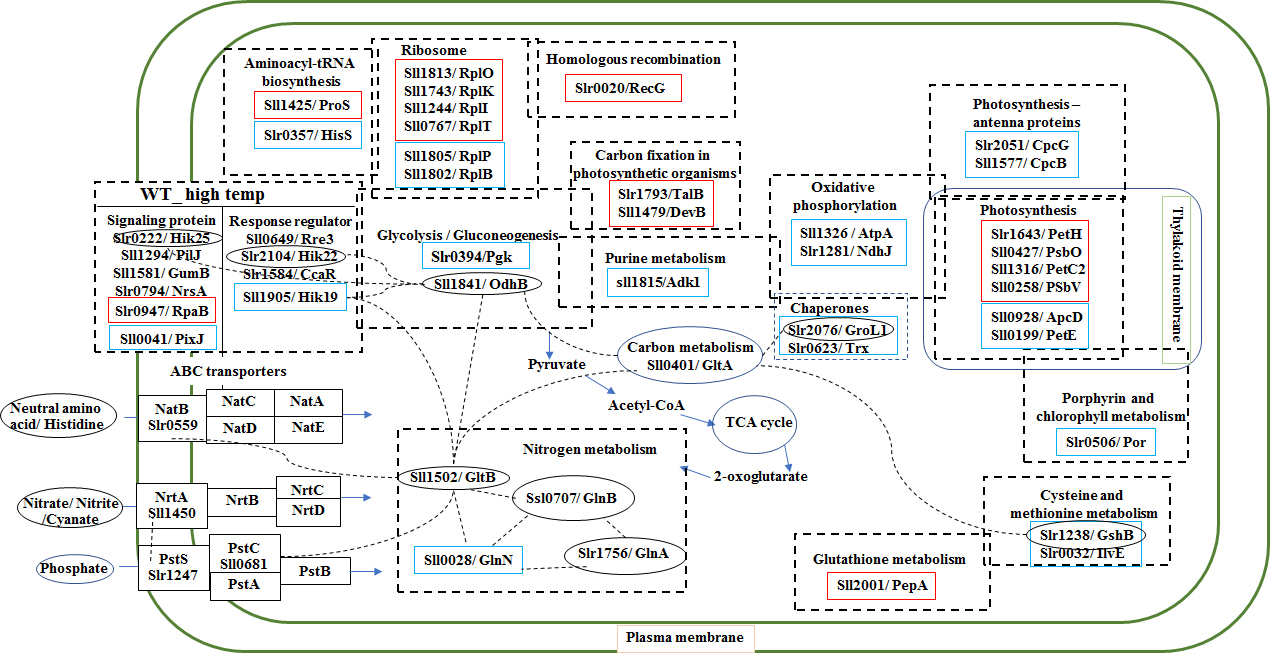


B


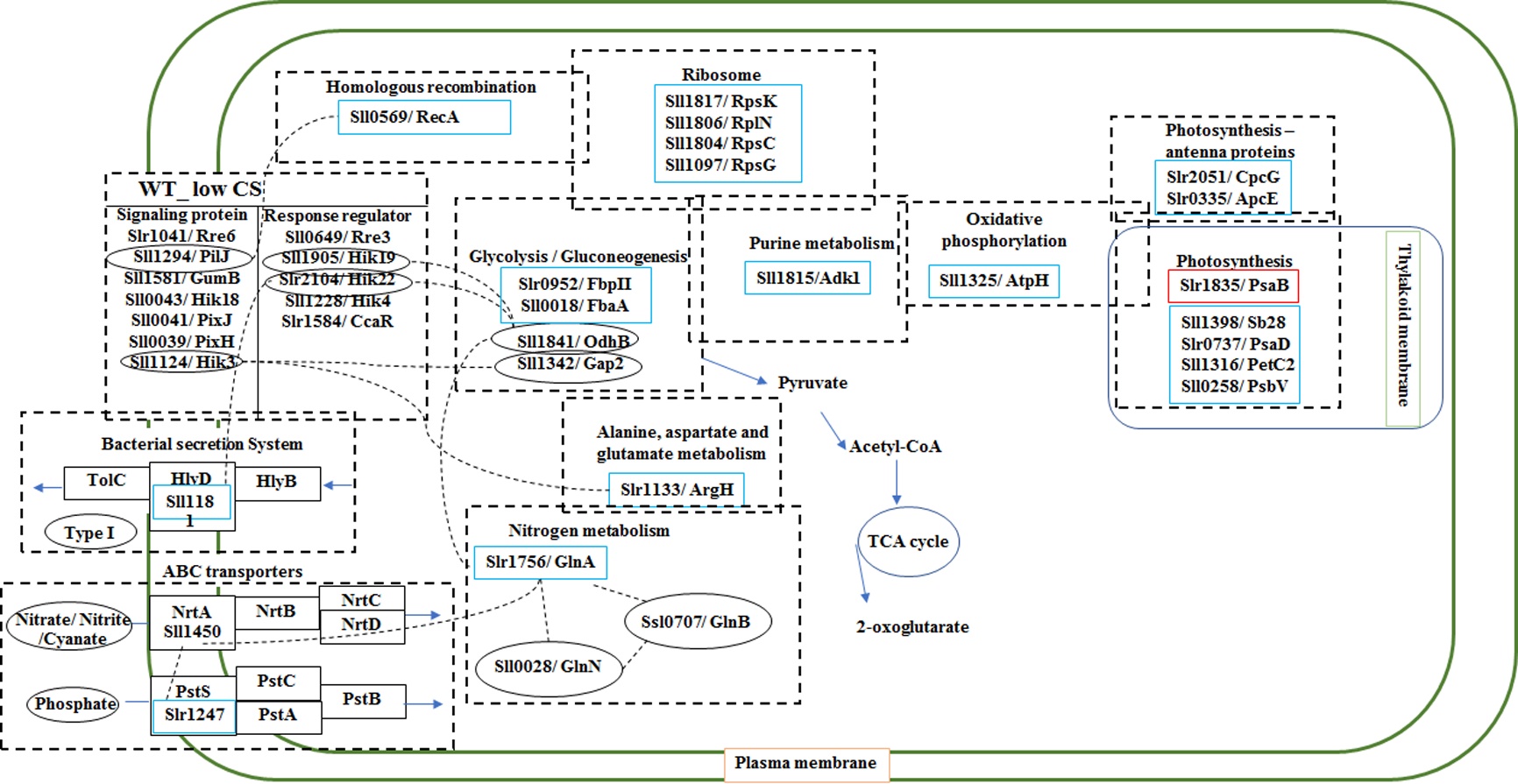


C


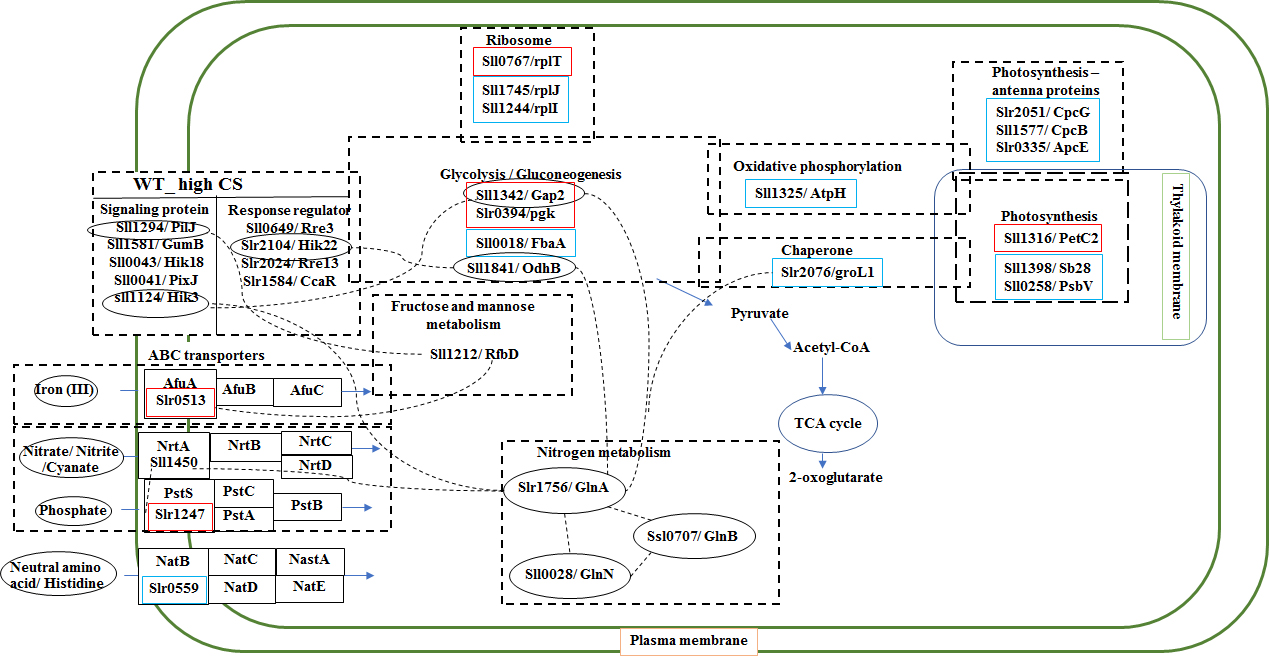


D

**Supplementary Figure 5** The protein-protein interaction (PPI) network of the two-component system, their response regulators and the up- and downregulated protein found in *Synechocystis* sp PCC6803 WT strain under (A) low temperature stress, (B) high temperature stress, (C) combined stress (CS) of low temperature and nitrogen depletion and (D) combined stress (CS) of high temperature and nitrogen depletion. The PPI network of the regulated proteins in metabolic pathways was illustrated in the dotted-line-boxes. The up- and downregulated proteins were in red and blue boxes, respectively. The dotted lines represent interactions between a protein in ovals. **Note:** Hik28 was found in *Arthrospira platensis* strain C1 under low temperature stress (Kurdrid et al., BMC Molecular and Cell Biology, 2020).
