# Supplementary material for: Hik28-dependent and Hik28-independent ABC transporters were revealed by proteome-wide analysis of ΔHik28 under combined stress
Source: BMC Mol Cell Biol. 2022 Jul 6;23:27. doi: 10.1186/s12860-022-00421-w (PMC9258054; doi:10.1186/s12860-022-00421-w)
Supplement: Supplementary file 13 — Additional file 13. [file 12860_2022_421_MOESM13_ESM.docx]

**Supplementary Figure 6** The PPI subnetwork constructed by using STRING version 11.5 (<https://string-db.org/cgi/input?sessionId=bXwea5WPVDuX&input_page_show_search=off>) of (A) the hypothetical proteins found in upstream (Slr0516) and downstream (Sll0493 and Slr0517) of *Synechocystis*-Hik28, two-component system and ABC transporter. The PPI subnetwork of two-component system, ABC transporter and their client proteins in *Synechocystis-*MT and WT strains under (B, F) low temperature stress, (C, G) high temperature stress, (D, H) combined stress of low temperature and nitrogen depletion, and (E, I) combined stress of high temperature and nitrogen depletion. The proteins in the group of ABC transporters were shown in dotted oval.

**
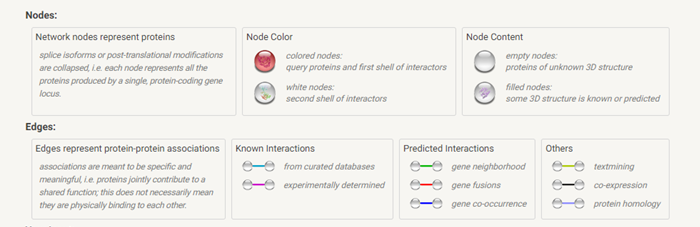
**

(A)


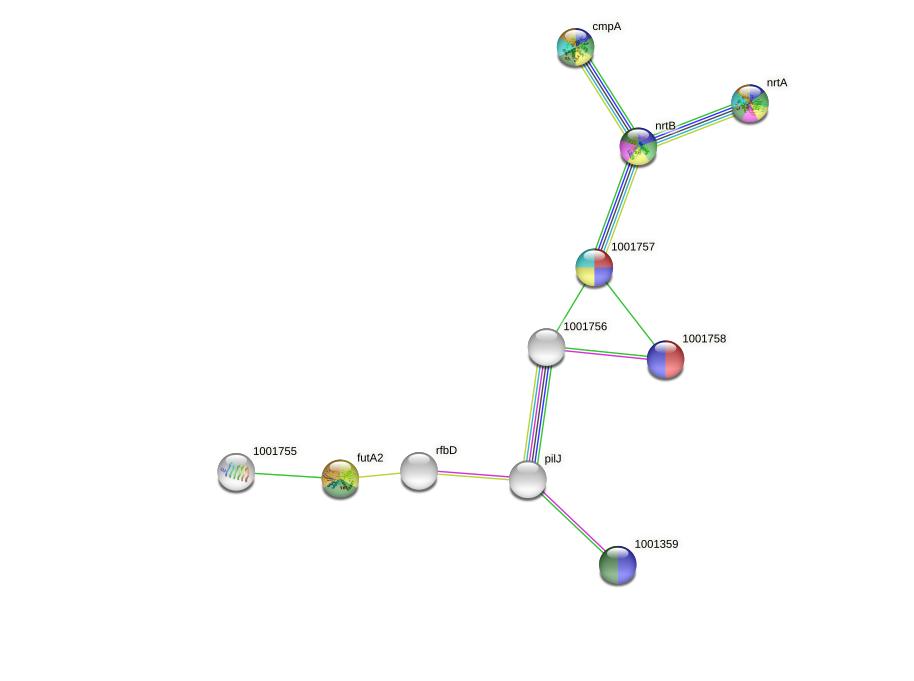


Sll0474 1001756 - sensory transduction histidine kinase Hik28

Sll1294 pilJ- methyl-accepting chemotaxis protein (MCP) homologue

Sll0473 1001757 - hypothetical protein

Slr0517 1001758 - hypothetical protein

Slr0516 1001755 - hypothetical protein

Sll0493 1001359 - hypothetical protein

Slr0040 cmpA - bicarbonate transporter

Sll1450 nrtA - Part of the ABC transporter complex NrtABCD involved in nitrate uptake

Sll1451 nrtB - Nitrate import permease protein nrtb; Part of the ABC transporter complex NrtABCD involved in nitrate uptake

Slr0513 futA2 - Fe(3+) ABC transporter substrate-binding protein

Sll1212 rfbD - GDP-D-mannose dehydratase

(B) MT_ low temp


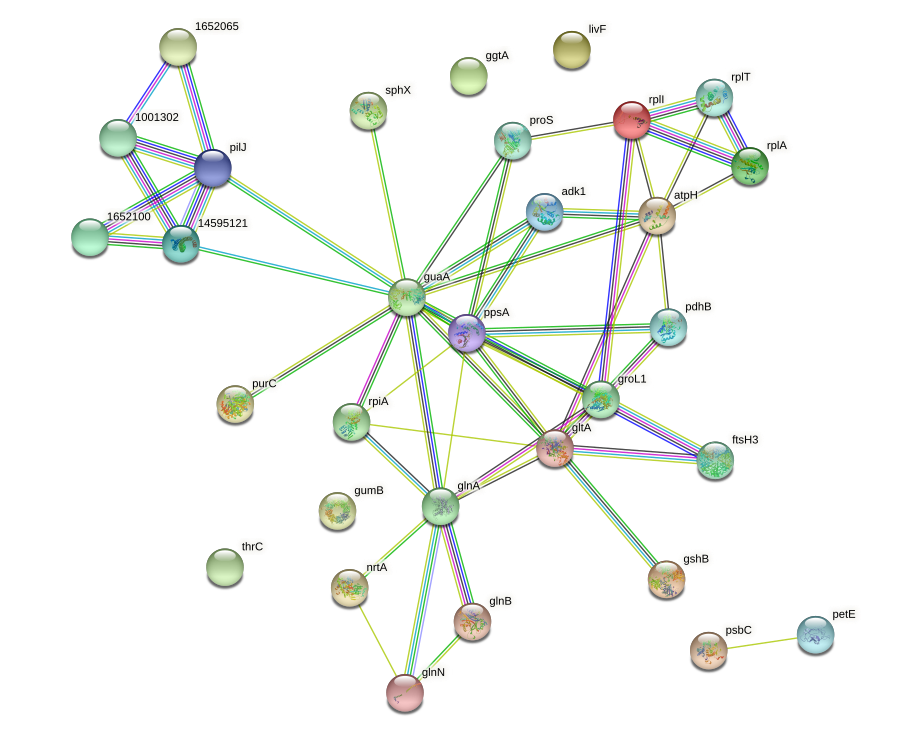


Sll1294 pilJ- methyl-accepting chemotaxis protein (MCP) homologue

Sll1581 gumB protein

Ssl0707 glnB-P-II family nitrogen regulator

Sll0041 14595121 - GAF domain-containing protein

Sll0039 1001302 -two-component system, chemotaxis family, response regulator PixH (downregulated)

Slr1041 1652100 - twitching motility two-component system response regulator PilG

Slr1594 1652065 - response regulator, PatA subfamily

Sll0679 sphX - Protein sphx; May be involved in the system for phosphate transport across the cytoplasmic membrane (downregulated)

Sll0374 livF- urea ABC transporter ATP-binding subunit UrtE(downregulated proteins)

Slr0747 ggtA - Atp-binding subunit of an abc-type osmolyte transporter; Belongs to the ABC transporter superfamily (downregulated)

Sll1450 nrtA - Part of the ABC transporter complex NrtABCD involved in nitrate uptake

Sll1425 proS - Proline--trna ligase; Catalyzes the attachment of proline to tRNA(Pro) in a two- step reaction: proline is first activated by ATP to form Pro-AMP and then transferred to the acceptor end of tRNA(Pro) (upregulated)

Slr1756 glnA - type I glutamate--ammonia ligase (downregulated)

Slr0288 glnN - Glutamate--ammonia ligase; Belongs to the glutamine synthetase family

Slr2076 groL1 - 60 kDa chaperonin 1(downregulated)

Slr1604 ftsH3 - Atp-dependent zinc metalloprotease ftsh3 (downregulated)

Slr1238 gshB - glutathione synthase (downregulated)

Sll1172 thrC- Threonine synthase; Catalyzes the gamma-elimination of phosphate from L- phosphohomoserine and the beta-addition of water to produce L- threonine. (upregulated)

Sll1721 pdhB - Pyruvate dehydrogenase e1 component subunit beta (downregulated)

Slr0301 ppsA - Phosphoenolpyruvate synthase; Catalyzes the phosphorylation of pyruvate to phosphoenolpyruvate (downregulated)

Sll1325 atpH - Atp synthase subunit delta; F(1)F(0) (upregulated)

Slr0194 rpiA - Ribose-5-phosphate isomerase A; Catalyzes the reversible conversion of ribose-5-phosphate to ribulose 5-phosphate (upregulated)

Sll0199 petE - Plastocyanin; Participates in electron transfer between P700 and the cytochrome b6-f complex in photosystem I (downregulated)

Sll0851 psbC - Photosystem ii cp43 reaction center protein (downregulated)

Sll1815 adk1 - Adenylate kinase 1 (downregulated)

Slr1226 purC - Phosphoribosylaminoimidazole-succinocarboxamide synthase (upregulated)

Sll1801 Rpl23-50S ribosomal protein L23 (downregulated)

Sll1244 rplI - 50S ribosomal protein L9; Binds to the 23S rRNA (upregulated)

Sll0767 rplT - 50S ribosomal protein L20 (downregulated)

Sll1744 rplA - 50S ribosomal protein L1 (upregulated)

Slr0213 guaA - Gmp synthase [glutamine-hydrolyzing]; Catalyzes the synthesis of GMP from XMP

Sll0401 gltA - citrate synthase

(C) MT_high temp


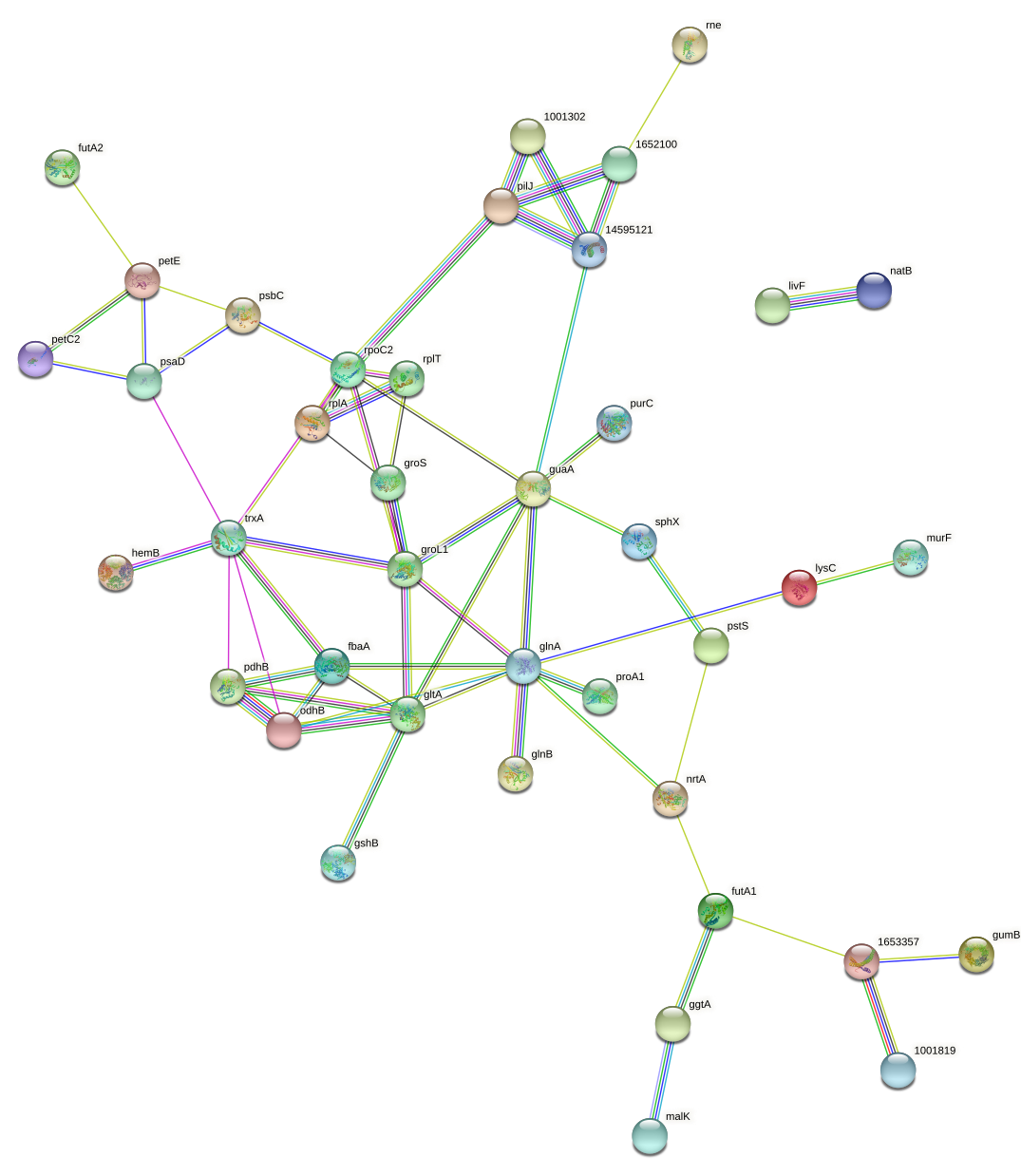


Sll1294 pilJ- methyl-accepting chemotaxis protein (MCP) homologue

Sll1581 gumB protein

Ssl0707 glnB-P-II family nitrogen regulator

Sll0041 14595121 - GAF domain-containing protein (upregulated)

Sll0039 1001302 -two-component system, chemotaxis family, response regulator PixH (upregulated)

Slr1041 1652100 -twitching motility two-component system response regulator PilG

Slr1594 1652065 -response regulator, PatA subfamily

Slr2104 1653081 -Hybrid sensory kinase, Hik22

Sll0396 1001644 -OmpR subfamily

Slr0794 1001819 - cation or drug efflux system protein

Slr1295 futA1 - Fe(3+) ABC transporter substrate-binding protein (downregulated)

Sll0680 pstS - phosphate transport system substrate-binding protein (downregulated)

Sll0679 sphX - PstS family phosphate ABC transporter substrate-binding protein (downregulated)

Sll0374 livF- urea ABC transporter ATP-binding subunit UrtE (downregulated)

Slr0747 ggtA - Atp-binding subunit of an abc-type osmolyte transporter; Belongs to the ABC transporter superfamily (downregulated)

Slr1224 malK-ABC transporter ATP-binding protein (downregulated)

Slr0559 natB-ABC transporter substrate-binding protein (upregulated)

Slr0513 futA2 - Fe(3+) ABC transporter substrate-binding protein (downregulated)

Sll0373 proA1-Gamma-glutamyl phosphate reductase 1 (downregulated)

Slr1756 glnA - type I glutamate--ammonia ligase (downregulated)

Slr2076 groL1-60 kDa chaperonin 1 (downregulated)

Slr2075 groS - 10 kDa chaperonin (upregulated)

Slr0623 trxA-component of the thioredoxin-thioredoxin reductase system (downregulated)

Slr1238 GshB - glutathione synthase (downregulated)

Slr0657 lysC-annotation not available (downregulated)

Sll0018 fbaA-Fructose-bisphosphate aldolase class 2 (upregulated)

Sll1841 odhB- dihydrolipoamide acetyltransferase component (E2) of pyruvate dehydrogenase complex (downregulated)

Sll1721 pdhB - Pyruvate dehydrogenase e1 component subunit beta (downregulated)

Slr1351 murF-Udp-n-acetylmuramoyl-tripeptide--d-alanyl-d-alanine ligase; Involved in cell wall formation (upregulated)

Sll1450 nrtA - Part of the ABC transporter complex NrtABCD involved in nitrate uptake (upregulated)

Sll0199 petE - Plastocyanin; Participates in electron transfer between P700 and the cytochrome b6-f complex in photosystem I (downregulated)

Slr0737 psaD - photosystem I reaction center subunit II (downregulated)

Sll1316 petC2 - cytochrome b6-f complex iron-sulfur subunit (upregulated)

Sll0851 psbC - Photosystem ii cp43 reaction center protein (downregulated)

Sll1994 hemB - porphobilinogen synthase (upregulated)

Slr1226 purC - Phosphoribosylaminoimidazole-succinocarboxamide synthase (upregulated)

Sll0767 rplT - 50S ribosomal protein L20 (upregulated)

Sll1744 rplA - 50S ribosomal protein L1 (upregulated)

Slr1129 rne -ribonuclease E (upregulated)

Sll1789 rpoC2 -RNA polymerase beta prime subunit (downregulated)

Sll0401 gltA - citrate synthase

Slr0213 guaA - Gmp synthase [glutamine-hydrolyzing]; Catalyzes the synthesis of GMP from XMP

Slr1270 1653357 -outer membrane factor, OMF family (upregulated)

(D) MT_low CS


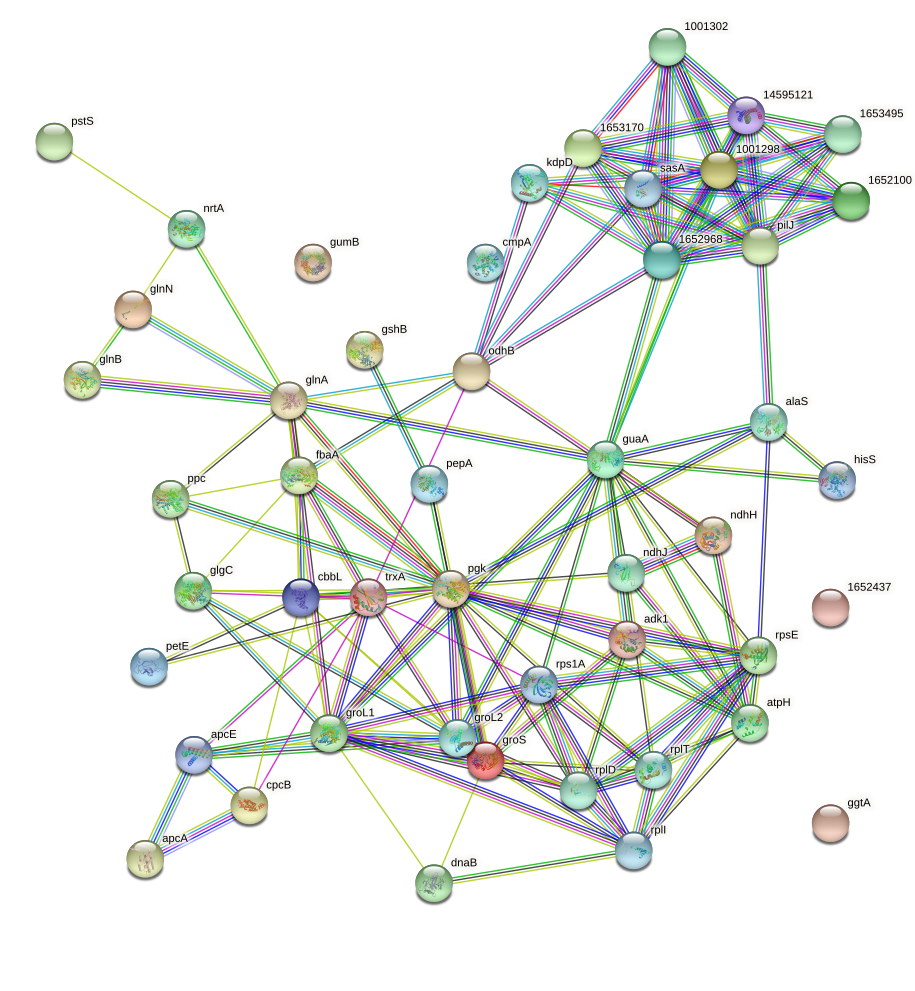


Slr1041 1652100 - twitching motility two-component system response regulator PilG

Sll1294 pilJ- methyl-accepting chemotaxis protein (MCP) homologue

Sll1581 gumB protein

Ssl0707 glnB-P-II family nitrogen regulator

Sll0041 14595121 - GAF domain-containing protein (downregulated)

Slr1731 kdpD - sensor histidine kinase

Sll0039 1001302 -two-component system, chemotaxis family, response regulator PixH

Slr0947 1653495 - two-component system, OmpR family, response regulator RpaB

Sll0750 SasA - two-component system, OmpR family, clock-associated histidine kinase

Sll0043 1001298 -two-component system, chemotaxis family, sensor histidine kinase and response regulator, Hik18

Sll1228 1653170 -two-component system, sensor histidine kinase and response regulator, Hik4

Sll1905 1652968 -two-component system, sensor histidine kinase and response regulator, Hik19 (downregulated)

Sll0680 pstS - phosphate transport system substrate-binding protein (downregulated)

Sll0240 ggtA - iron(III) transport system ATP-binding protein (downregulated)

Slr0040 cmpA -bicarbonate transporter (upregulated)

Sll1450 nrtA - Part of the ABC transporter complex NrtABCD involved in nitrate uptake

Slr0357 hisS - histidyl-tRNA synthetase (downregulated)

Sll0362 AlaS - alanyl-tRNA synthetase (upregulated)

Slr0288 glnN - Glutamate--ammonia ligase; Belongs to the glutamine synthetase family (downregulated)

Slr1756 glnA - type I glutamate--ammonia ligase

Slr2076 groL1 - 60 kDa chaperonin 1 (downregulated)

Slr2075 groS - 10 kDa chaperonin (downregulated)

Slr0623 trxA-component of the thioredoxin-thioredoxin reductase system (upregulated)

Sll0416 groEL-2 -60kD chaperonin 2 (downregulated)

Slr1238 gshB - glutathione synthase (upregulated)

Slr0833 dnaB -replicative DNA helicase (downregulated)

Sll2001 PepA -leucine aminopeptidase (upregulated)

Slr0394 pgk -phosphoglycerate kinase (downregulated)

Sll0018 fbaA-Fructose-bisphosphate aldolase class 2 (upregulated)

Slr0009 cbbL -ribulose-1,5-bisphosphate carboxylase/oxygenase large subunit (downregulated)

Slr0261 ndhH -NADH dehydrogenase subunit 7 (upregulated)

Slr1281 ndhJ -NADH dehydrogenase subunit I (downregulated)

Sll1325 atpH - Atp synthase subunit delta; F(1)F(0) (downregulated)

Sll0199 petE - Plastocyanin; Participates in electron transfer between P700 and the cytochrome b6-f complex in photosystem I (upregulated)

Sll1580 cpcC -phycocyanin associated linker protein (downregulated)

Sll1577 cpcB -phycocyanin b subunit (downregulated)

Slr0335 apcE -phycobilisome LCM core-membrane linker polypeptide

Slr2067 apcA -allophycocyanin a chain (downregulated)

Sll1815 adk1 - Adenylate kinase 1 (upregulated)

Sll0920 ppc -phosphoenolpyruvate carboxylase (upregulated)

Slr1901 1652437 - ABC transporter (downregulated)

Sll1812 rps5 -30S ribosomal protein S5 (upregulated)

Sll1800 rplD - 50S ribosomal protein L4 (downregulated)

Slr1356 rps1A -30S ribosomal protein S1 (downregulated)

Sll1244 rplI - 50S ribosomal protein L9; Binds to the 23S rRNA (upregulated)

Sll0767 rplT - 50S ribosomal protein L20 (upregulated)

Slr1176 glgC -ADP-glucose pyrophosphorylase (downregulated)

Slr1270 outer membrane factor, OMF family (upregulated)

Sll1841 odhB- dihydrolipoamide acetyltransferase component (E2) of pyruvate dehydrogenase complex

Slr0213 guaA - Gmp synthase [glutamine-hydrolyzing]; Catalyzes the synthesis of GMP from XMP

(E) MT_high CS


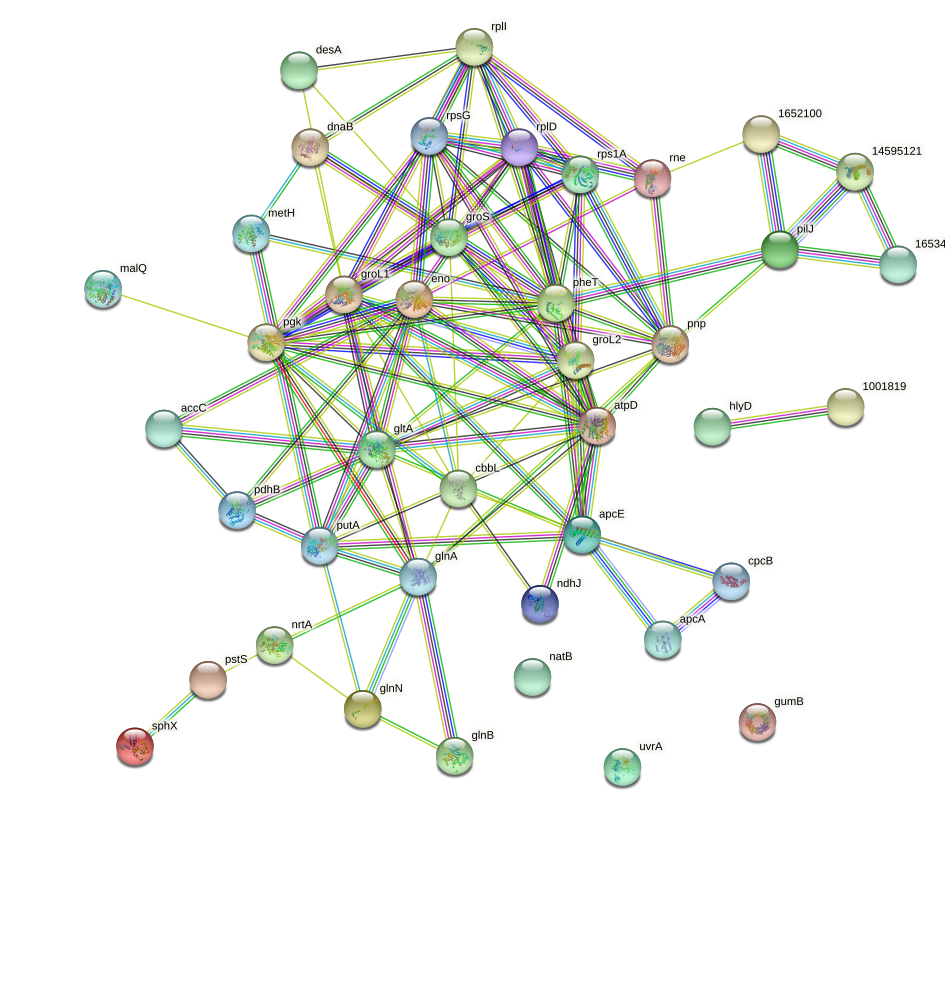


Slr1041 1652100 - twitching motility two-component system response regulator PilG0

Sll1294 pilJ- methyl-accepting chemotaxis protein (MCP) homologue

Sll1581 gumB protein

Ssl0707 glnB-P-II family nitrogen regulator (upregulated)

Sll0041 14595121 - GAF domain-containing protein (upregulated)

Slr0947 1653495 - two-component system, OmpR family, response regulator RpaB

Slr1350 desA -fatty acid desaturase

Slr0794 1001819 - cation or drug efflux system protein

Sll0649 1652041 - two-component system, OmpR family, response regulator

Slr2024 1652347 - CheY subfamily

Sll0679 sphX - PstS family phosphate ABC transporter substrate-binding protein (downregulated)

Slr0559 natB-ABC transporter substrate-binding protein (upregulated)

Sll0680 pstS - phosphate transport system substrate-binding protein

Sll1450 nrtA - Part of the ABC transporter complex NrtABCD involved in nitrate uptake (downregulated)

Sll1561 putA -delta-1-pyrroline-5-carboxylate dehydrogenase (downregulated)

Sll1553 pheT - phenylalanyl-tRNA synthetase (upregulated)

Sll1181 hlyD -HlyD family of secretion proteins (downregulated)

Slr2076 groL1 - 60 kDa chaperonin 1 (downregulated)

Slr2075 groS - 10 kDa chaperonin (downregulated)

Sll0416 groEL-2 -60kD chaperonin 2 (downregulated)

Slr0212 metH -5-methyltetrahydrofolate--homocysteine methyltransferase (upregulated)

Slr0833 dnaB -replicative DNA helicase (downregulated)

Sll0053 accC -biotin carboxylase (downregulated)

Slr0752 eno- enolase (upregulated)

Slr0394 pgk -phosphoglycerate kinase (downregulated)

Sll1721 pdhB - Pyruvate dehydrogenase e1 component subunit beta (upregulated)

Slr0009 cbbL -ribulose-1,5-bisphosphate carboxylase/oxygenase large subunit (downregulated)

Slr1844 uvrA -excinuclease ABC subunit A (downregulated)

Slr1329 atpB -ATP synthase b subunit (upregulated)

Slr1281 ndhJ -NADH dehydrogenase subunit I (downregulated)

Sll1194 psbU -photosystem II 12 kD extrinsic protein (upregulated)

Sll0258 psbV -cytochrome c550 (upregulated)

Sll1580 cpcC -phycocyanin associated linker protein (downregulated)

Sll1577 cpcB -phycocyanin b subunitB (downregulated)

Slr0335 apcE -phycobilisome LCM core-membrane linker polypeptide (downregulated)

Slr2067 apcA -allophycocyanin a chain (downregulated)

Sll1800 rplD - 50S ribosomal protein L4 (downregulated)

Slr1356 rps1A -30S ribosomal protein S1 (downregulated)

Sll1097 rpsG - 30S ribosomal protein S7 (upregulated)

Sll1244 rplI - 50S ribosomal protein L9; Binds to the 23S rRNA (upregulated)

Sll1043 pnp -polyribonucleotide (upregulated)

Slr1129 rne -ribonuclease E (downregulated)

Sll1676 malQ - 4-alpha-glucanotransferase (upregulated)

Slr1756 glnA - type I glutamate--ammonia ligase

Sll0401 gltA - citrate synthase

(F) WT_low temp


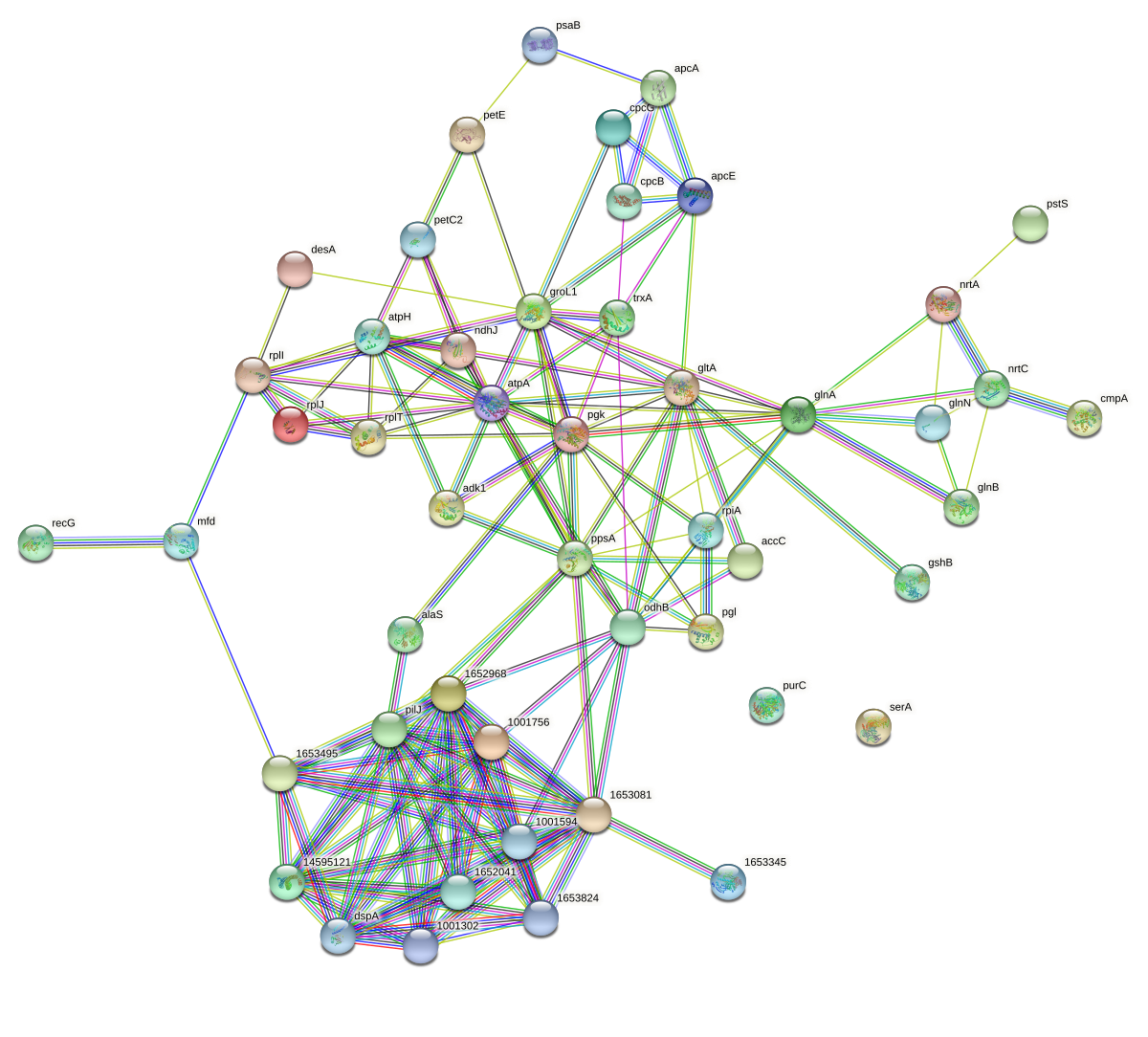


Ssl0707 glnB-P-II family nitrogen regulator

Slr0222 1001594 -hybrid sensory kinase Hik25

Sll0041 14595121 - GAF domain-containing protein

Sll1294 pilJ- methyl-accepting chemotaxis protein (MCP) homologue

Slr0947 1653495 - two-component system, OmpR family, response regulator RpaB

Sll0039 1001302 -two-component system, chemotaxis family, response regulator PixH

Sll0698 dspA - two-component system, OmpR family, sensor histidine kinase Hik33

Slr1350 desA - fatty acid desaturase

Sll1371 1653345 - catabolite gene activator protein

Sll0474 1001756 - sensory transduction histidine kinase Hik28

Sll0649 1652041 - two-component system, OmpR family, response regulator

Sll1905 1652968 - two-component system, sensor histidine kinase and response regulator Hik19

Slr2104 1653081 - Hybrid sensory kinase, Hik22

Slr1584 1653824 - OmpR subfamily

Sll0474 1001756 - sensory transduction histidine kinase Hik28

Slr1247 pstS - periplasmic phosphate binding protein (downregulated)

Slr0040 cmpA - bicarbonate transporter (upregulated)

Sll1450 nrtA - Part of the ABC transporter complex NrtABCD involved in nitrate uptake

Sll1452 nrtC - nitrate transport protein

Sll0362 AlaS - alanyl-tRNA synthetase (downregulated)

Slr0623 trxA- component of the thioredoxin-thioredoxin reductase system (upregulated)

Slr2076 groL1 - 60 kDa chaperonin 1 (downregulated)

Slr1238 gshB - glutathione synthase (downregulated)

Sll0053 accC - biotin carboxylase (upregulated)

Sll1908 serA - phosphoglycerate dehydrogenase (downregulated)

Slr0394 pgk - phosphoglycerate kinase (downregulated)

Slr0301 ppsA - Phosphoenolpyruvate synthase; Catalyzes the phosphorylation of pyruvate to phosphoenolpyruvate (downregulated)

Slr0020 recG - DNA recombinase (upregulated)

Sll0377 mfd - transcription-repair coupling factor (downregulated)

Sll1326 atpA - ATP synthase a subunit (downregulated)

Sll1325 atpH - Atp synthase subunit delta; F(1)F(0) (downregulated)

Slr1281 ndhJ - NADH dehydrogenase subunit I (downregulated)

Sll1479 pgl - glucose-6-P-dehydrogenase (upregulated)

Slr0194 rpia - Ribose-5-phosphate isomerase A; Catalyzes the reversible conversion of ribose-5-phosphate to ribulose 5-phosphate (upregulated)

Slr1835 psaB - P700 apoprotein subunit Ib (upregulated)

Sll1316 petC2 - cytochrome b6-f complex iron-sulfur subunit (downregulated)

Sll0199 petE - Plastocyanin; Participates in electron transfer between P700 and the cytochrome b6-f complex in photosystem I (upregulated)

Slr2051 cpcG - phycobilisome rod-core linker polypeptide (downregulated)

Sll1577 cpcB - phycocyanin b subunit (downregulated)

Slr0335 apcE - phycobilisome LCM core-membrane linker polypeptide (downregulated)

Slr2067 apcA - allophycocyanin a chain (downregulated)

Sll1815 adk1 - Adenylate kinase 1 (upregulated)

Slr1226 purC - Phosphoribosylaminoimidazole-succinocarboxamide synthase (upregulated)

Sll1745 rplJ - 50S ribosomal protein L10 (downregulated)

Sll1244 rplI - 50S ribosomal protein L9; Binds to the 23S rRNA (upregulated)

Sll0767 rplT - 50S ribosomal protein L20 (downregulated)

Sll1841 odhB- dihydrolipoamide acetyltransferase component (E2) of pyruvate dehydrogenase complex

Sll0401 gltA - citrate synthase

Slr1756 glnA - type I glutamate--ammonia ligase

Slr0288 glnN - Glutamate--ammonia ligase; Belongs to the glutamine synthetase family

(G) WT_high temp


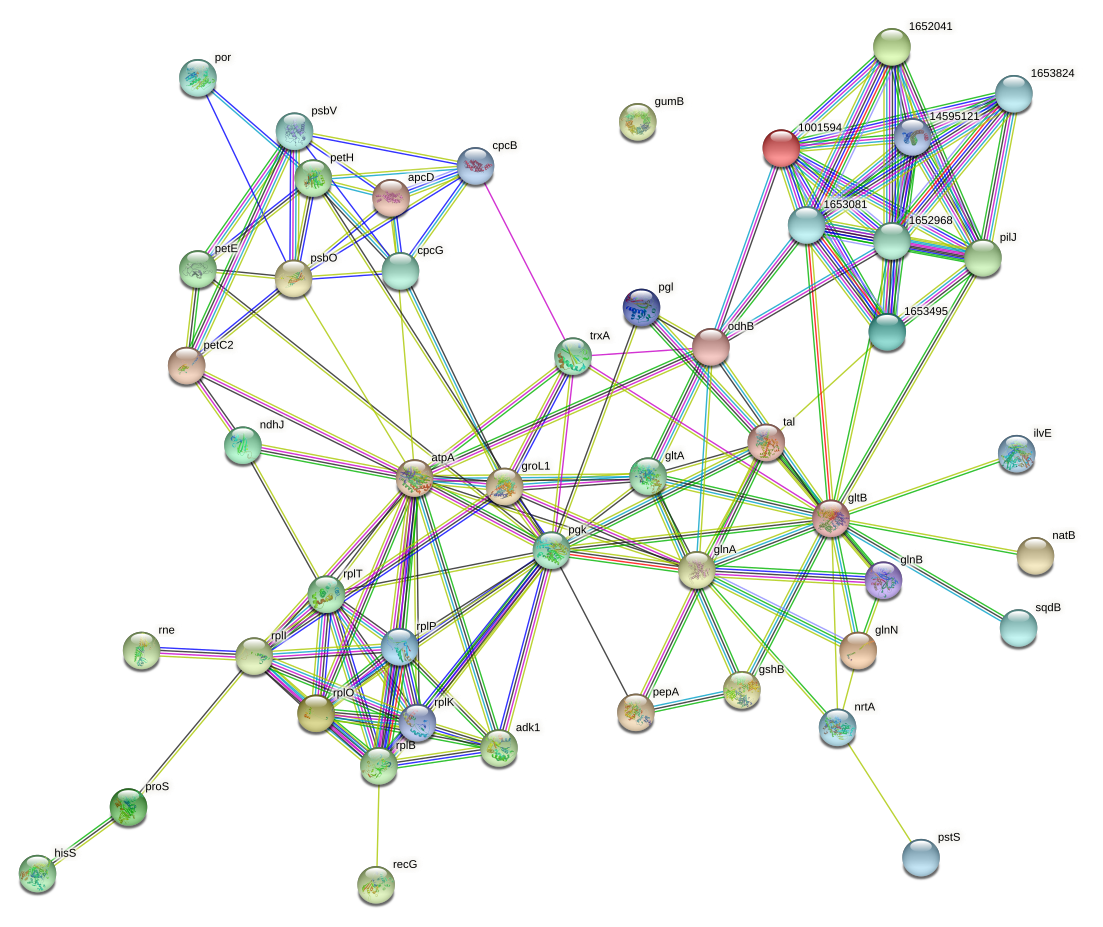


Ssl0707 glnB-P-II family nitrogen regulator

Slr0222 1001594 -hybrid sensory kinase Hik25

Sll0041 14595121 - GAF domain-containing protein (downregulated)

Sll1294 pilJ- methyl-accepting chemotaxis protein (MCP) homologue

Slr0947 1653495 - two-component system, OmpR family, response regulator RpaB

Sll1581 gumB protein

Sll5060 two-component hybrid sensor and regulator

Sll0649 1652041 - two-component system, OmpR family, response regulator

Sll1905 1652968 -two-component system, sensor histidine kinase and response regulator Hik19 (downregulated)

Slr2104 1653081 -Hybrid sensory kinase Hik22

Slr1584 1653824 - OmpR subfamily

Sll1450 nrtA - Part of the ABC transporter complex NrtABCD involved in nitrate uptake

Slr1247 pstS - periplasmic phosphate binding protein

Slr0559 natB-ABC transporter substrate-binding protein

Slr1020 sqdB - sulfolipid biosynthesis protein (upregulated)

Sll1425 proS - Proline--tRNA ligase; Catalyzes the attachment of proline to tRNA(Pro) in a two- step reaction: proline is first activated by ATP to form Pro-AMP and then transferred to the acceptor end of tRNA(Pro) (upregulated)

Slr0357 hisS - histidyl-tRNA synthetase (downregulated)

Slr0288 glnN - Glutamate--ammonia ligase; Belongs to the glutamine synthetase family (downregulated)

Slr0623 trxA-component of the thioredoxin-thioredoxin reductase system (downregulated)

Slr2076 groL1 - 60 kDa chaperonin 1 (downregulated)

Slr0032 ilvE -branched-chain amino acid aminotransferase (downregulated)

Slr1238 gshB - glutathione synthase (downregulated)

Sll2001 pepA -leucine aminopeptidase (upregulated)

Slr0394 pgk -phosphoglycerate kinase (downregulated)

Slr0020 recG - DNA recombinase (upregulated)

Sll1326 atpA - ATP synthase a subunit (downregulated)

Slr1281 ndhJ -NADH dehydrogenase subunit I (downregulated)

Slr1793 tal – transaldolase (upregulated)

Sll1479 pgl - glucose-6-P-dehydrogenase (upregulated)

Sll0928 apcD - allophycocyanin-B (downregulated)

Slr1643 petH - ferredoxin-NADP oxidoreductase (upregulated)

Sll0427 psbO -photosystem II manganese-stabilizing polypeptide (upregulated)

Sll1316 petC2 - cytochrome b6-f complex iron-sulfur subunit (upregulated)

Sll0258 psbV -cytochrome c550 (upregulated)

Sll0199 petE - Plastocyanin; Participates in electron transfer between P700 and the cytochrome b6-f complex in photosystem I (downregulated)

Slr2051 cpcG - phycobilisome rod-core linker polypeptide (downregulated)

Sll1577 cpcB -phycocyanin b subunit (downregulated)

Slr0506 por - protochlorophyllide oxido-reductase (downregulated)

Sll1815 adk1 - Adenylate kinase 1 (downregulated)

Sll1813 rplO -50S ribosomal protein L15 (upregulated)

Sll1805 rplP - 50S ribosomal protein L16 (downregulated)

Sll1802 rplB - 50S ribosomal protein L2 (downregulated)

Sll1743 rplK - 50S ribosomal protein L11 (upregulated)

Sll1244 rplI - 50S ribosomal protein L9; Binds to the 23S rRNA (upregulated)

Sll0767 rplT - 50S ribosomal protein L20 (upregulated)

Slr1129 rne -ribonuclease E (upregulated)

Sll1502 gltB - glutamate synthase (ferredoxin)

Slr1756 glnA - type I glutamate--ammonia ligase

Sll0401 gltA - citrate synthase

Sll1841 odhB- dihydrolipoamide acetyltransferase component (E2) of pyruvate dehydrogenase complex

(H) WT_low CS


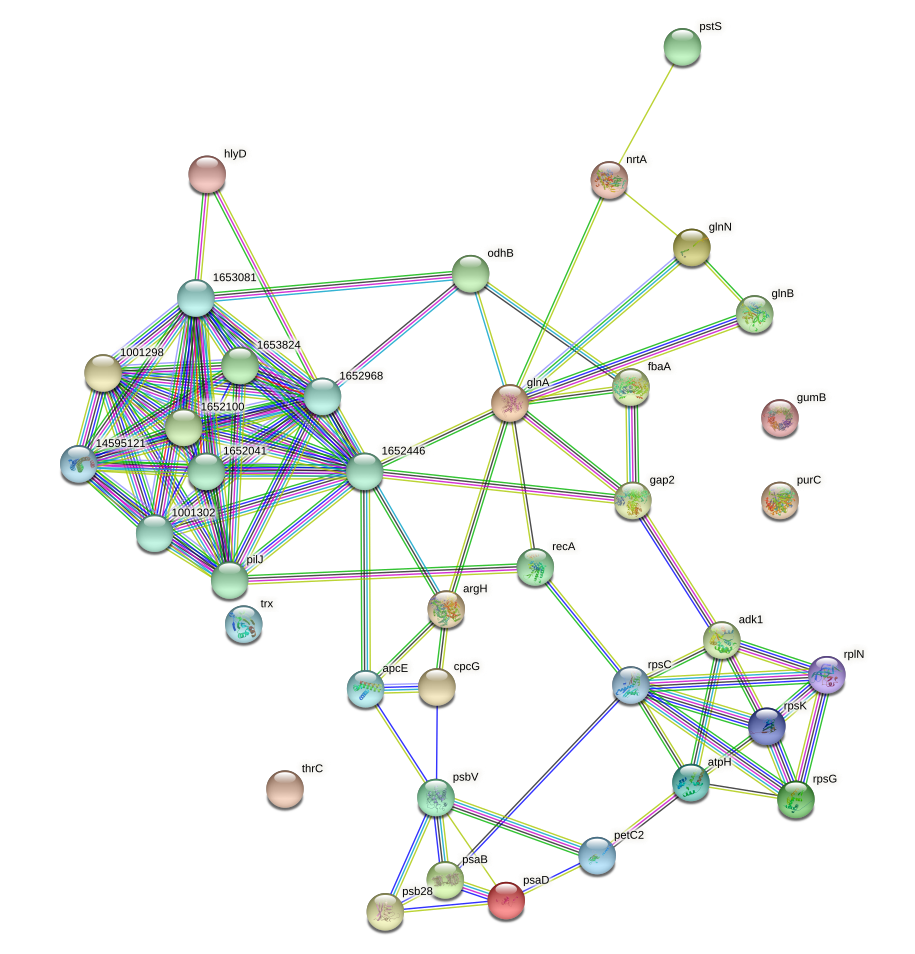


Slr1041 1652100 - twitching motility two-component system response regulator PilG

Sll1294 pilJ- methyl-accepting chemotaxis protein (MCP) homologue

Sll1581 gumB protein

Ssl0707 glnB-P-II family nitrogen regulator

Sll0043 1001298 -two-component system, chemotaxis family, sensor histidine kinase and response regulator, Hik18

Sll0041 14595121 - GAF domain-containing protein

Sll0039 1001302 -two-component system, chemotaxis family, response regulator PixH

Sll1124 1652446 - sensory transduction histidine kinase PlpA/Hik3

Sll5060 two-component hybrid sensor and regulator

Sll0649 1652041 - two-component system, OmpR family, response regulator

Sll1905 1652968 -two-component system, sensor histidine kinase and response regulator Hik19

Slr2104 1653081 -Hybrid sensory kinase Hik22

Slr1584 1653824 - OmpR subfamily

Slr1247 pstS -periplasmic phosphate binding protein (upregulated)

Sll1450 nrtA - Part of the ABC transporter complex NrtABCD involved in nitrate uptake

Slr1133 argH - L-argininosuccinate lyase (downregulated)

Slr1756 glnA - type I glutamate--ammonia ligase (downregulated)

Sll1181 hlyD -HlyD family of secretion proteins (downregulated)

Sll0033 crtH (downregulated)

Slr0623 trxA-component of the thioredoxin-thioredoxin reductase system (upregulated)

Sll1172 thrC- Threonine synthase; Catalyzes the gamma-elimination of phosphate from L- phosphohomoserine and the beta-addition of water to produce L- threonine (downregulated)

Slr0952 fbpII - fructose 1,6-bisphosphatase (downregulated)

Sll1342 gap2 - glyceraldehyde-3-phosphate dehydrogenase (NADP+) (phosphorylating) (downregulated)

Sll0018 fbaA-Fructose-bisphosphate aldolase class 2 (downregulated)

Sll0569 recA - RecA gene product (downregulated)

Sll1325 atpH - Atp synthase subunit delta; F(1)F(0) (downregulated)

Sll1398 psb13-photosystem II 13 kD protein (downregulated)

Slr1835 psaB - P700 apoprotein subunit Ib (upregulated)

Slr0737 psaD - photosystem I reaction center subunit II (downregulated)

Sll1316 petC2 - cytochrome b6-f complex iron-sulfur subunit (downregulated)

Sll0258 psbV -cytochrome c550 (downregulated)

Slr2051 cpcG - phycobilisome rod-core linker polypeptide (downregulated)

Slr0335 apcE -phycobilisome LCM core-membrane linker polypeptide (downregulated)

Sll1815 adk1 - Adenylate kinase 1 (downregulated)

Slr1226 purC - Phosphoribosylaminoimidazole-succinocarboxamide synthase (upregulated)

Sll1817 rpsK - 30S ribosomal protein S11 (downregulated)

Sll1806 rplN - 50S ribosomal protein L14 (downregulated)

Sll1804 rpsC - 30S ribosomal protein S3 (downregulated)

Sll1097 rpsG - 30S ribosomal protein S7 (downregulated)

Slr0288 glnN - Glutamate--ammonia ligase; Belongs to the glutamine synthetase family

Sll1841 odhB- dihydrolipoamide acetyltransferase component (E2) of pyruvate dehydrogenase complex

Sll1342 gap2 - glyceraldehyde-3-phosphate dehydrogenase (NADP+) (phosphorylating)

(I) WT-high CS


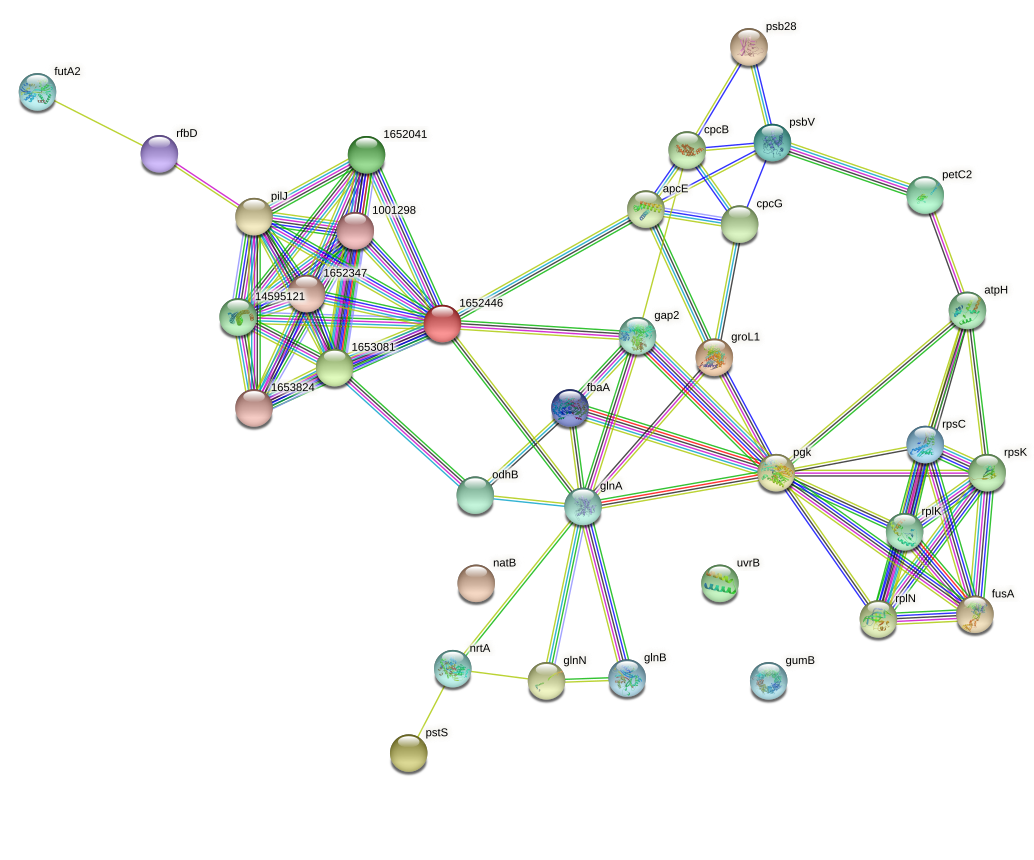


Sll1294 pilJ- methyl-accepting chemotaxis protein (MCP) homologue

Sll1581 gumB protein

Ssl0707 glnB-P-II family nitrogen regulator

Sll0043 1001298 -two-component system, chemotaxis family, sensor histidine kinase and response regulator Hik18

Sll0041 14595121 - GAF domain-containing protein

Sll1124 1652446 - sensory transduction histidine kinase PlpA/Hik3

Sll5060 two-component hybrid sensor and regulator

Sll0649 1652041 - two-component system, OmpR family, response regulator

Slr2104 1653081 -Hybrid sensory kinase Hik22

Slr2024 1652347 - CheY subfamily

Slr1584 1653824 - OmpR subfamily

Slr1247 pstS -periplasmic phosphate binding protein (upregulated)

Slr0559 natB-ABC transporter substrate-binding protein (downregulated)

Slr0513 futA2 - Fe(3+) ABC transporter substrate-binding protein (upregulated)

Sll1450 nrtA - Part of the ABC transporter complex NrtABCD involved in nitrate uptake

Slr2076 groL1 - 60 kDa chaperonin 1 (downregulated)

Sll1342 gap2 - glyceraldehyde-3-phosphate dehydrogenase (NADP+) (phosphorylating) (upregulated)

Slr0394 pgk -phosphoglycerate kinase (upregulated)

Sll0018 fbaA-Fructose-bisphosphate aldolase class 2 (downregulated)

Sll0459 uvrB - excinuclease ABC subunit B (downregulated)

Sll1325 atpH - Atp synthase subunit delta; F(1)F(0) (downregulated)

Sll1398 psb13 -photosystem II 13 kD protein (downregulated)

Sll1316 petC2 - cytochrome b6-f complex iron-sulfur subunit (upregulated)

Sll0258 psbV -cytochrome c550 (downregulated)

Slr2051 cpcG - phycobilisome rod-core linker polypeptide (downregulated)

Sll1577 cpcB -phycocyanin b subunit (downregulated)

Slr0335 apcE -phycobilisome LCM core-membrane linker polypeptide (downregulated)

Sll1817 rpsK - 30S ribosomal protein S11 (downregulated)

Sll1806 rplN - 50S ribosomal protein L14 (downregulated)

Sll1804 rpsC - 30S ribosomal protein S3 (upregulated)

Sll1743 rplK - 50S ribosomal protein L11(upregulated)

Slr1756 glnA - type I glutamate--ammonia ligase

Slr0288 glnN - Glutamate--ammonia ligase; Belongs to the glutamine synthetase family

Sll1212 rfbD - GDP-D-mannose dehydratase

Sll1841 odhB- dihydrolipoamide acetyltransferase component (E2) of pyruvate dehydrogenase complex
